# Inferring gene expression profiles of tumor clones

**DOI:** 10.1093/bioinformatics/btag717

**Published:** 2026-10-03

**Authors:** Shadi Shafighi, Barbara Jurzysta, Ewa Szczurek

**Affiliations:** Faculty of Mathematics, Informatics and Mechanics, University of Warsaw, Warsaw, 02-097, Poland; Faculty of Mathematics, Informatics and Mechanics, University of Warsaw, Warsaw, 02-097, Poland; Faculty of Mathematics, Informatics and Mechanics, University of Warsaw, Warsaw, 02-097, Poland; Institute of AI for Health, Helmholtz Zentrum Munchen, Neuherberg, 85764, Germany

## Abstract

**Motivation:**

Tumors are heterogeneous mixtures of clonal subpopulations of cells that co-evolve in time and space. Apart from genetic differences, cancer clones may reside in different locations in the tumor tissue and show various transcriptional phenotypes, with specific gene expression profiles and marker genes. Computational tools that simultaneously infer genotypes and transcriptional phenotypes of clones in tumor tissues are lacking.

**Results:**

We introduce ClonalGE, a probabilistic graphical model identifying cancer clone genotypes, their gene expression profiles, and spatial mapping within tumor tissue. The model proceeds by probabilistic matching of single nucleotide variant sites in spatial transcriptomics to clone-specific variants inferred from DNA sequencing data, and by deconvoluting gene expression in spatial transcriptomics spots into contributions from individual clones. We propose a procedure that utilizes the Monte Carlo posterior samples for identification of differentially expressed genes between clones, using a Highest Density Interval plus Region of Practical Equivalence (HDI+ROPE) criterion that jointly requires credible and practically meaningful effect sizes. ClonalGE demonstrates superior performance on simulated data. Applied to a prostate cancer sample, it reveals clone-specific expression patterns and outperforms its predecessor model in accurately inferring gene expression profiles and explaining spatial transcriptomics signals. Our approach advances the functional and phenotypic characterization of clonal cell subpopulations in tumors.

**Availability:**

The implementation of ClonalGE and the data used in this study can be accessed in the following GitHub repository https://github.com/szczurek-lab/ClonalGE/. The benchmarking data alongside the intermediate results are available on Zenodo at DOI: 10.5281/zenodo.20446991.

## 1 Introduction

Tumor cells evolve through the acquisition of genetic alterations, leading to the formation of subpopulations of cells known as clones. Targeted cancer therapies often fail to eradicate all of the clones, thereby exerting selective evolutionary pressure on the remaining cells (Greaves and Maley 2012). At the same time, cancer cells exhibit plasticity on the transcriptomic level, which allows different tumor cells to switch states and adapt to their microenvironment (Yuan *et al.* 2019). Understanding the heterogeneity of a tumor, particularly in terms of the variation in transcription phenotype and localization of the different clones, is critical for effectively targeting and eliminating the cancerous cells while minimizing the risk of recurrence (Meacham and Morrison 2013).

Previous cancer heterogeneity analyses primarily focused on clonal genotypes (Tarabichi *et al.* 2021). These approaches infer the evolutionary history of somatic alterations in the tumor from bulk DNA sequencing data (Roth *et al.* 2014, Beerenwinkel *et al.* 2015, 2016, Deshwar *et al.* 2015, Jiang *et al.* 2016, Vandin 2017), multi-region bulk DNA sequencing (De Bruin *et al.* 2014), or single cell DNA sequencing (Kang *et al.* 2022, 2025, Markowska *et al.* 2022). Such methods can indicate the number of the clones, their frequencies and genotypes in the sample. However, they cannot estimate clonal gene expression profiles, nor identify genes that are differentially expressed between the clones, nor localize in high resolution where the clones are in the tumor tissue.

The association between gene expression and clones was studied by Kim *et al.* (2015) using semi-supervised identification of clones based on single cell RNA sequencing (scRNA-seq) data. This involved analyzing the gene expression and activated pathways of each clone. An alternative approach predicted gene expression of copy number variant (CNV)-based clones using previously learned global correlation of CNV and gene expression from multiple scRNA-seq datasets and corresponding bulk DNA sequencing (DNA-seq) data (Müller *et al.* 2018). Campbell *et al.* (2019) proposed an approach that assigned gene expression states to cancer clones using RNA-seq and DNA-seq for single cells independently sampled from a heterogeneous tumor population. Other methods, such as Cardelino (McCarthy *et al.* 2020), CACTUS (Shafighi *et al.* 2021) and CaClust (Oksza-Orzechowski *et al.* 2024), combined DNA-seq and scRNA-seq data to first infer cancer clones and their genotypes from DNA-seq and then map single cells to the clones based on the shared single nucleotide variants (SNVs). By aggregating RNA signal from the single cells into clones, these approaches identified clone-specific phenotypes. However, these methods were unable to identify the localization of the clones in the tissue and required availability of single cell RNA-sequencing data.

Recent spatial transcriptomics (ST) technology measures gene expression in a spatially resolved manner (Ståhl *et al.* 2016, Cheng *et al.* 2023). One of the popular ST protocols, 10X Genomics Visum, utilizes the RNA sequencing protocol and generates a mixture of gene expression profiles from multiple cells present in given localized spots. ST can be seen as an alternative source of gene expression measurement for identifying clonal expression profiles simultaneously with their localization. Several methods emerged that combine signal in DNA-seq and ST data to identify cancer clones and their mapping in the tissue based on CNVs (Elyanow *et al.* 2021, Erickson *et al.* 2022, Wu *et al.* 2025). These methods, however, ignore important evolutionary events caused by SNVs, which play a crucial role in tumor progression and treatment.

To address this issue, recently, we proposed a model called Tumoroscope (Shafighi *et al.* 2024), which combines DNA-seq and ST data alongside H&E images to infer and localize clones based on SNVs. The main functionality of Tumoroscope is inference of the proportions of each ST spot occupied by each clone. Estimation of clonal gene expression profiles in the Tumoroscope framework is done as a post-processing step, using linear regression with weights set to the inferred proportions (Shafighi *et al.* 2024). Therefore, the accuracy of the gene expression profile estimation using Tumoroscope is limited. Moreover, the framework does not include the ability to perform differential gene expression analysis. This is due to the fact that Tumoroscope followed by linear regression does not estimate the full distribution of clone-specific gene expression profiles, but rather provides its point estimates, making it impossible to perform differential gene expression analyses. Therefore, an approach that integrates DNA-seq and ST data by a single model, focusing on gene expression analysis of the clones, identification of their gene expression distributions, differentially expressed genes, and explaining the ST measurements as compositions of gene expression stemming from the localized clones, is lacking.

In this study, we present ClonalGE, a novel statistical graphical model that infers clone-specific gene expression and the composition of clones present in localized spots within a tissue sample. ClonalGE integrates DNA-seq, H&E images and spatial transcriptomics data, by probabilistic matching of read counts for somatic SNVs shared between DNA-seq and ST and by identifying cancerous regions and counts of cells in spots from H&E images. The model leverages expression measurements of genes across the tissue sample to improve the accuracy of mapping clones to specific regions. Furthermore, ClonalGE enables the generation of distributions of clone-specific gene expression profiles, allowing for subsequent differential expression analysis between pairs of clones. Our model contributes to the increased understanding of tumor heterogeneity, not only on genetic but also on the transcriptional and spatial level.

## 2 Methods

### 2.1 ClonalGE framework overview

We propose a novel probabilistic framework for inference of gene expression profiles of cancer clones, alongside the spatial mapping of the clones across the tissue and estimation of differential gene expression between the clones, using H&E stained images, spatially-resolved transcriptomics, and bulk DNA-seq. This framework takes as input pre-processed data, including: genotypes and frequencies of the inferred clones (based on bulk DNA-seq data; Fig. 1a), the total and alternated read counts over selected mutations (based on ST reads; Fig. 1b), the gene expression profile of the spots (based on ST reads; Fig. 1b), and regions of the tissue annotated as cancer and the number of cells in the annotated ST spots (based on H&E images; Fig. 1c).

**Figure 1:**
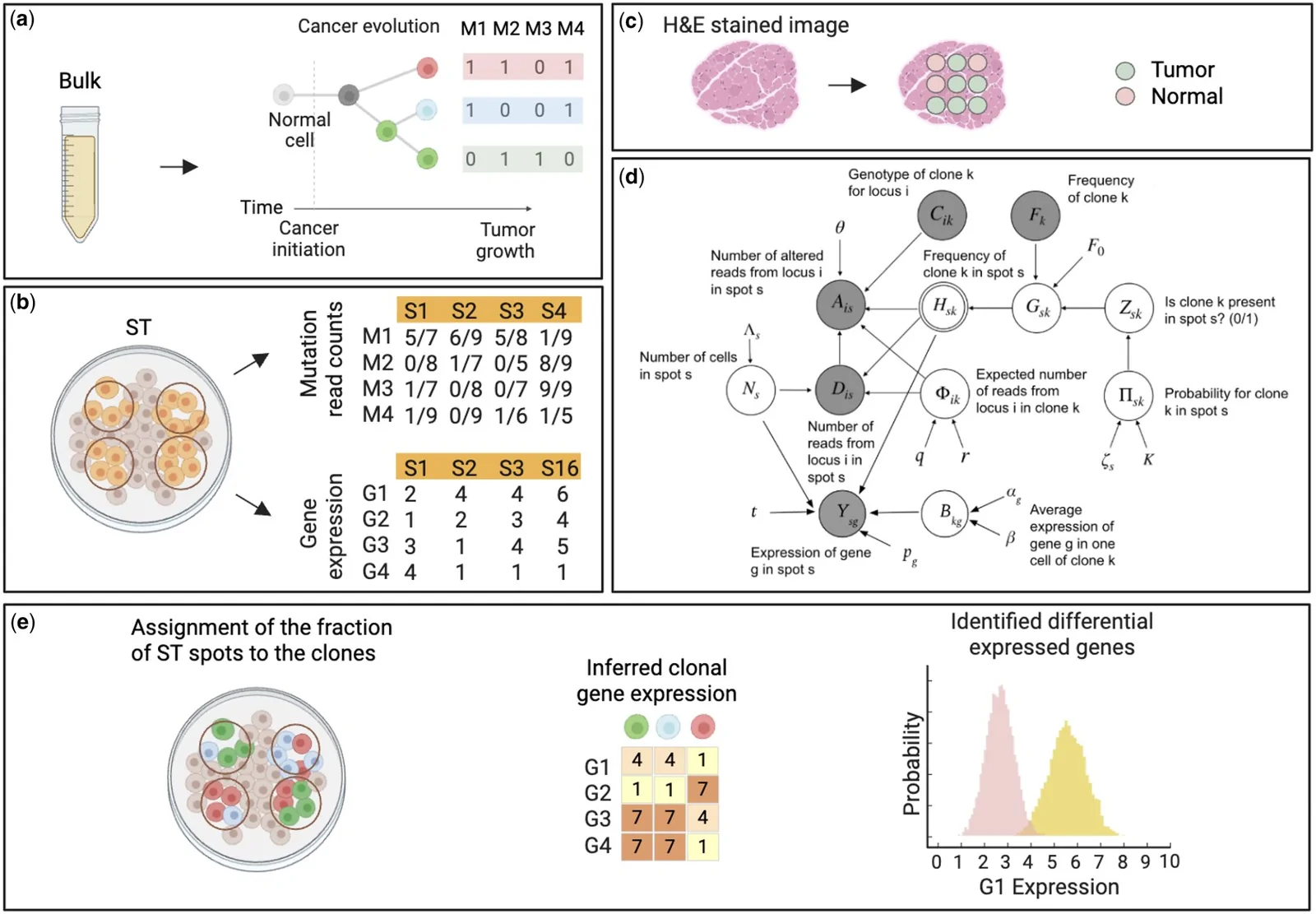
ClonalGE framework overview. (a–c) Data preprocessing. Tumor clones, their genotypes and frequencies are found from DNA-seq data (a). Alternated to total readcounts at the SNV sites and gene expression measurements for genes in the cancerous spots are identified in ST data (b). Cancerous regions are identified and initial cell counts are found from H&E images of the tumor tissue (c). (d) ClonalGE probabilistic model; a complete list of all variables and parameters is provided in Supplementary Table S1. (e) Outputs: Inferred fraction of the clones in the spots, inferred clonal gene expression, and identified differentially expressed genes. The figure is generated by Biorender.

The core of the framework is a probabilistic graphical model called ClonalGE. ClonalGE combines inference of clonal gene expression and composition of ST spots. The model is a profound extension of our previous model, Tumoroscope (Shafighi *et al.* 2024), by incorporating additional variables for the gene expression in the spots. The assumptions behind this extension are that each clone has its specific average gene expression profile; and that the expression of the genes observed at the spots is a mixture of the gene expressions in the clones that are present in those spots. For inference, we proceed in two stages. Tumoroscope is run first to provide initialisation of all the hidden variables to ClonalGE. In the second stage, we use Metropolis Hastings within Gibbs for estimation of ClonalGE (Fig. 1d). The model returns the fraction of each annotated ST spot occupied by each clone together with the gene expression profile of each clone (Fig. 1e). Finally, we use the model-inferred distributions of gene expression for each clone to perform differential gene expression analysis between clones (Fig. 1e). The details of input data and each step of the methodology are described in the following subsections.

### 2.2 Prostate cancer sample

We used a published prostate cancer dataset including DNA-seq, H&E images, and ST data (Berglund *et al.* 2018). The DNA-seq was performed on tissue layers adjacent to ST and H&E. The data was available for twelve sections covering half of the prostate extracted by a prostatectomy on a patient with adenocarcinoma (Gs 3 + 4, pT3b, PSA 7.1). Besides, we used the DNA-seq data from the patient’s blood to serve as a control for detecting the somatic mutations. The data generation protocol as well as data availability were described by Berglund *et al.* (2018). Details of data preprocessing for ClonalGE follow the procedure of Shafighi *et al.* (2024) and are briefly covered in the following sections.

### 2.3 Annotation of cancerous spots and counting cells in spots from H&E images

For cancerous spot identification, the regions containing cancer cells were annotated by an expert pathologist taking advantage of the H&E image of each section. Afterward, using a custom script in QuPath (Bankhead *et al.* 2017), the spots inside the annotated regions were identified. These spots were located in three of the twelve original sections [named SP1, SP2 and SP3 as in Shafighi *et al.* (2024)].

Cells in the cancerous spots were counted using another custom script in QuPath. The script accepts input in the form of coordinates and diameters of spots to define target regions. Subsequently, QuPath’s built-in algorithm is utilized to detect and enumerate nuclei. The algorithm’s parameters were optimized by matching the output counts with manual cell count within randomly selected spots.

### 2.4 Somatic mutation calling from DNA-seq data

We called somatic point mutations using Vardict (Lai *et al.* 2016). The final set of mutations contained variants called with a *P*-value threshold equal to 0.1 in at least one of the DNA-seq sections (i.e. the set of mutations was the union over mutations found in individual sections). Initially, 2104 somatic mutations were called across all sections. After filtering for presence in both DNA-seq and ST data, 280 mutations remained and were used for downstream analysis.

### 2.5 Extraction of read count data for somatic mutations that were detected both in DNA-seq and ST data

We selected the mutations for which at least one alternated read in at least one section was found. Calculation of the the total and alternated reads over the mutations in ST data was done by a script provided by Shafighi *et al.* (2024), which finds the selected DNA-seq mutations in the ST bam files and counts the corresponding mapped reads. We next gave the alternated and total read counts in DNA-seq data as input to the method of phylogenetic tree inference. Finally, we gave the alternated and total read counts in ST data for the same mutations as input to the ClonalGE model.

### 2.6 Estimation of the genotypes and frequencies of the clones from DNA-seq data

The genotypes and frequencies of the clones were obtained using a statistical method for inference of phylogenetic trees called Canopy (Jiang *et al.* 2016). Canopy takes the following inputs: (i) variant allele frequencies (VAF) of somatic single nucleotide alterations, obtained by Vardict (Lai *et al.* 2016) and (ii) ratios of the coverage between the tumor and normal sample for somatic copy number alterations (CNAs), obtained by FalconX (Chen *et al.* 2017). We used the multi-sample feature of the Canopy to infer the clonal evolution across the sections. Clone 1 returned by Canopy should be considered normal (without any somatic mutations).

### 2.7 ClonalGE

ClonalGE is a probabilistic graphical model for estimating the gene expression profile of the clones alongside with the fraction of the clones in the ST spots across the tumor sample.

Let k∈{1,…,K} index the clones derived from DNA sequencing data and i∈{1,…,I} index the loci with somatic point mutations observed both in ST and DNA-sequencing data. $F_{k}$ indicates the frequency of occurrence of clone *k* in the whole sample. $C_{ik}$ codes the inferred genotype, taking values between 0 and 1, and is defined by the ratio of the number of alleles of clone *k* carrying a mutation on locus *i* to the total number of alleles. Both the frequencies $F_{k}$ and genotypes $C_{ik}$ are specifications of the clones estimated from DNA-seq data and are considered as observed variables in the model.

Let s∈{1,…,S} index the spots in the ST data. $Z_{sk}$ is a hidden binary variable, indicating the presence of clone *k* in spot *s*. It follows a Bernoulli distribution with success parameter $\Pi_{sk}$, following a Beta prior distribution.


$$P(Z_{sk}|\Pi_{sk})∼\text{Bern}(\Pi_{sk}),$$

$$P(\Pi_{sk}|\zeta_{s},K)∼\text{Beta}\left(\frac{\zeta_{s}}{K},1\right).$$

The hyperparameter $\zeta_{s}$ influences expected number of clones in a spot, which is given by


$$\frac{\zeta_{s}}{\zeta_{s}+K}.$$

Depending on the indicator variables $Z_{sk}$ and the observed clone genotypes and frequencies, we expect higher or lower values of unnormalized abundances of the clones in the spots. These unnormalized abundances for each spot *s* and clone *k* are described in the model using Gamma-distributed hidden variables$G_{sk}:$


$$P(G_{sk}|F_{k},F_{0},Z_{sk})∼\text{Gamma}(f{\left(F_{k}\right)}^{Z_{sk}}F_{0}{\;}^{1-Z_{sk}},1).$$

The first parameter of the Gamma distribution is calculated using a discretized value of $F_{k}$. The discretization is performed for technical reasons and helps avoid numerically too small values. Specifically, when clone frequencies $F_{k}$ are very small, the shape parameter of the Gamma distribution for $G_{sk}$ approaches zero, leading to numerically degenerate distributions where nearly all probability mass concentrates at zero. The discretization ensures a minimum effective shape parameter, preserving numerical stability while maintaining the relative ordering of clone frequencies. The discretization level of 0.05 was chosen to balance numerical stability with the resolution of frequency differences. The discretization function *f* is defined by $f(x)=\left\lceil \frac{x}{0.05}\right\rceil \cdot 100$. The hyperparameter $F_{0}$ is used as a pseudo-count for clones not assigned to the spot, indicated by $Z_{sk}=0$, and is set to a small number.

$H_{sk}$ is a deterministic variable that can be interpreted as a fraction of spot *s* assigned to clone *k*. It follows a Dirichlet distribution and is calculated based on the unnormalized abundances $G_{sk}$ as


$$H_{sk}=\frac{G_{sk}}{\sum_{l=1}^{K}G_{sl}}.$$

Note that $H_{sk}$’s are one of the two central sets of random variables in the model—they solve the task of identifying the proportions of the clones in the spots.

$\Phi_{ik}$ is a latent variable that is interpreted as an average read count at position *i* per one cell of clone *k*. It follows a Gamma distribution with hyperparameters *r* and *q*:


$$P(\Phi_{ik}|r,q)∼\text{Gamma}(r,q).$$

The number of cells in spot *s*, represented by a variable $N_{s}$ is modeled with a Poisson distribution:


$$P(N_{s}|\Lambda_{s})∼\text{Poiss}(\Lambda_{s}),$$

where $\Lambda_{s}$ is the provided at input cell count at spot *s*, estimated from H&E images.

$D_{is}$ represents counts of reads over locus *i* coming from spot *s* and $A_{is}$ indicates how many of them are mutated (altered). $D_{is}$ and $A_{is}$ are observed variables extracted from ST reads and are given to the model and assumed to follow a Poisson distribution and a Binomial distribution respectively:


$$P(D_{is}|H_{s.},\Phi_{i.},N_{s})∼\text{Pois}\left(N_{s}\sum_{k}H_{sk}\Phi_{ik}\right),$$

$$P(A_{is}|D_{is},\Phi_{i.},H_{s.},C_{i.})∼\text{Binom}\left(D_{is},\frac{\sum_{k}\Phi_{ik}C_{ik}H_{sk}}{\sum_{k}\Phi_{ik}H_{sk}}\right).$$

All the above definitions concern also the previous model Tumoroscope by Shafighi *et al.* (2024). We now explain the model extension to account for gene expression values in the spot, allowing to estimate gene expression profiles of clones as an integral part of the model.

Let g∈{1,…,G} index the genes observed in the ST data. Our main goal is to estimate the average gene expression of gene *g* per one cell in clone *k*, represented by a hidden random variable $B_{kg}$, which is the second central variable in the model and is assumed to follow a Gamma distribution with hyperparameters $\alpha_{g}$ and β


$$P(B_{kg}|\alpha_{g},\beta )∼\text{Gamma}(\alpha_{g},\beta ).$$

An observed variable $Y_{sg}$ signifies the expression of gene *g* in spot *s* which is the main measurement in ST data and is given to the model at input. We assume a Negative Binomial distribution over $Y_{sg}$ with expected value of $N_{s}\sum_{k}H_{sk}B_{kg}$, which is the clonal gene expression multiplied by the number of cells of each clone in spot *s*. To overcome the batch effect observed when integrating the data from different sections, we added a scaling parameter $t_{j}$ to the model (with *j* indexing the sections and $t_{j}$ assumed to be common for all spots in section *j*). Then the expected value for $Y_{sg}$ is $t_{j}\cdot N_{s}\sum_{k}H_{sk}B_{kg}$ given that spot *s* is in section *j*. Considering $r_{sg}$ and $p_{g}$ as the parameters of the Negative Binomial distribution over $Y_{sg}$, the expected value of $Y_{sg}$ is $E[Y_{sg}]=\frac{(1-p_{g})r_{sg}}{p_{g}}$ and the variance is equal to $\mathrm{Var}(Y_{sg})=\frac{(1-p_{g})r_{sg}}{p_{g}^{2}}$. Using the formula for $E[Y_{sg}]$ we substitute $r_{sg}$ by


$$r_{sg}=\frac{E[Y_{sg}]p_{g}}{1-p_{g}},$$

which results in a useful reparametrization:


(1) $$Y_{sg}∼\text{NB}\left(\frac{E[Y_{sg}]p_{g}}{1-p_{g}},p_{g}\right).$$

This reparameterization is advantageous because it expresses the distribution directly in terms of the expected gene expression $E[Y_{sg}]$, which can be linked to the biologically meaningful clone-weighted expression contributions $N_{s}\sum_{k}H_{sk}B_{kg}$, while retaining the overdispersion properties of the Negative Binomial distribution appropriate for count data. Using this parametrization, we can rewrite the distribution of $Y_{sg}$:


$$P(Y_{sg}|t_{j},B_{.g},p_{g},H_{s.},N_{s})\;∼\;\text{NB}\left(t_{j}N_{s}\sum_{k}H_{sk}B_{kg}\frac{p_{g}}{1-p_{g}},p_{g}\right),$$

where spot *s* is in section *j*.

### 2.8 Metropolis-Hasting inside Gibbs sampling

For estimating the posterior distribution of the hidden variables in our probabilistic model, we iteratively generate samples from each hidden variable’s conditional distribution, given the remaining variables. We use a Metropolis-Hasting step inside the Gibbs sampler in case a closed-form distribution for sampling a random variable does not exist.

The sampling procedure for variables $\Phi_{ik}$, $Z_{sk}$ and $\Pi_{sk}$ remain the same as is described in Shafighi *et al.* (2024). Here we describe the sampling procedure for the remaining part of the model.

We use Metropolis-Hastings inside Gibbs sampler for variables $G_{sk}$, $N_{s}$, and $B_{kg}$ since there exists no closed form of their full conditional distributions. We choose a Truncated Normal distribution for the proposal distribution due to the non-negativity of these variables.

The proximity of the new sample from the current one, which is interpreted as the step size, is determined by the variance of the proposal distribution. During the sampling steps, we learn the optimal variance value for each variable as described by Shafighi *et al.* (2024).

Conditional distribution for $G_{sk}$:


$$\begin{array}{c} P(G_{sk}|F_{k},F_{0},Z_{sk},A_{is},D_{is})\\ \propto P(G_{sk}|F_{k},F_{0},Z_{sk})\prod_{i}P(D_{is}|\Phi_{i.},H_{s.},N_{s})\\ \times \prod_{i}P(A_{is}|D_{is},\Phi_{i.},H_{s.},C_{i.})\\ \times \prod_{g}P(Y_{sg}|t,B_{.g},p_{g},H_{s.},N_{s})\\ =\mathrm{Gamma}(f{\left(F_{k}\right)}^{Z_{sk}}F_{0}^{1-Z_{sk}},1)\prod_{i}Pois(N_{s}\sum_{k}H_{sk}\Phi_{ik})\\ \times \prod_{i}Bi\mathrm{nom}(D_{is},\frac{\sum_{k}\Phi_{ik}C_{ik}H_{sk}}{\sum_{k}\Phi_{ik}H_{sk}})\\ \times \prod_{g}NB(tN_{s}\sum_{k}H_{sk}B_{kg}\frac{p_{g}}{1-p_{g}},p_{g}), \end{array}$$

where with italic names we write the density functions of the respective probability distributions.

Conditional distribution for $N_{s}$:


$$\begin{array}{c} P(N_{s}|\Lambda_{s},D_{is},Y_{sg})\propto P(N_{s}|\Lambda_{s})\\ \times \prod_{i}P(D_{is}|\Phi_{i.},H_{s.},N_{s})\prod_{g}P(Y_{sg}|t,B_{.g},p_{g},H_{s.},N_{s})\\ =\mathrm{Pois}(\Lambda_{s})\prod_{i}Pois(N_{s}\sum_{k}H_{sk}\Phi_{ik})\\ \times \prod_{g}NB(tN_{s}\sum_{k}H_{sk}B_{kg}\frac{p_{g}}{1-p_{g}},p_{g}). \end{array}$$

Conditional distribution for $B_{kg}$:


$$\begin{array}{c} P(B_{kg}|\alpha_{g},\beta ,Y_{sg})\propto P(B_{kg}|\alpha_{g},\beta )\prod_{s}P(Y_{sg}|t,B_{.g},p_{g},H_{s.},N_{s})\\ =\mathrm{Gamma}(\alpha_{g},\beta )\prod_{s}NB(tN_{s}\sum_{k}H_{sk}B_{kg}\frac{p_{g}}{1-p_{g}},p_{g}). \end{array}$$

### 2.9 Hyper-parameter estimation and initialization of random variable values prior to MCMC

We estimate the hyper-parameters of the model based on the real data. First, the value of $\Lambda_{s}$ is set to the estimated number of cells in spot *s*, calculated based on the H&E images. Second, having a Negative Binomial distribution over $Y_{sg}$ with the success probability $p_{g}$, we have


$$p_{g}=\frac{E[Y_{sg}]}{\mathrm{Var}(Y_{sg})}.$$

Thus, we approximate $p_{g}$ by estimating $E[Y_{sg}]$ and $\mathrm{Var}(Y_{sg})$ using arithmetic mean and the variance of the given gene expression data for gene *g* over the spots.

For estimating the gene expression scaling factor, $t_{j}$, we calculate the mean value of $Y_{sg}$ for spots *s* that are present in section *j*. Let $m_{1},\ldots ,m_{J}$ be the average gene expression for spots in the *J* analyzed sections and let $m=\frac{1}{J}(m_{1}+\cdots +m_{J})$. Then for section *j* the estimated scaling factor is ${\hat{t}}_{j}=\frac{m_{j}}{m}$.

For estimating the hyperparameters $\alpha_{g}$ and β of the Gamma distribution of the $B_{kg}$ variables we use the formulas for expected value, $E[B_{kg}]=\alpha_{g}\beta$, and the variance, $\mathrm{Var}(B_{kg})=\alpha_{g}\beta^{2}$ of the Gamma distribution. Therefore, one could obtain β by dividing the two values.


$$\beta =\frac{\mathrm{Var}(B_{kg})}{E[B_{kg}]}.$$

Since we do not have *B* values as they are latent variables, we approximate them by the average expression of the gene per cell over clones:


Bkg≈∑k=1KBkgK≈1S·J∑j∑s in section j(YsgtjNs).

Therefore, we estimate $E[B_{kg}]$ and $\mathrm{Var}(B_{kg})$ by calculating the mean and variance of these average values across genes. By considering the average over clones, we are underestimating the variance and accordingly the values of β. Therefore, we multiply the estimated variance by a correction parameter $\omega_{B}$. The value of $\omega_{B}$ was estimated based on simulations and fixed to $\omega_{B}=2$.

Next, based on the expected value of the Gamma distribution over $B_{kg}$, $E[B_{kg}]=\alpha_{g}\beta$, we approximate $\alpha_{g}$ using the estimated $\hat{\beta }$ and the already calculated average expression of the genes over the clones.

We estimate the hyper-parameters of Gamma distribution over Φ, *r* and *q*, similarly to the $\alpha_{g}$ and β, using the approximation of the expected value and variance of Φ.


$$\hat{E}(\Phi )=\frac{\sum_{i}\sum_{s}\frac{D_{is}}{N_{s}}}{I\times S}$$

$$\hat{\mathrm{Var}}(\Phi )=\frac{\sum_{i}\sum_{s}(\frac{D_{is}}{N_{s}}-E(\Phi ))}{I\times S}.$$

Then, we estimate the hyper-parameters with


$$\hat{r}=\omega_{\phi }\frac{\hat{\mathrm{Var}}(\Phi )}{\hat{E}(\Phi )}\;\text{and}\;\hat{\text{q}}=\frac{\hat{\text{E}}(\Phi )}{\hat{\text{r}}}.$$

Here, we use a constant, $\omega_{\phi }$, for compensation of the underestimation of the *r*. This correction constant was again estimated based on simulations and fixed to $\omega_{\phi }=2$. The robustness of the inference to perturbations of the estimated hyperparameters $\alpha_{g}$, β, and $p_{g}$ is assessed in Supplementary Fig. S7.

To find initial values of hidden variables prior to MCMC, we first run Tumoroscope for at least 7000 iterations stopping after reaching the Geweke’s convergence criterion (Geweke *et al.* 1991), with the upper bound of 15 000 iterations. Afterward, we use the inferred variables to initialize the corresponding hidden variables in ClonalGE. We further use the regression model proposed in Shafighi *et al.* (2024) to initialize the *B* matrix.

### 2.10 Examining convergence

We examine the convergence of the MCMC for ClonalGE by using Geweke’s convergence diagnostic on the *H* variable matrix. We consider different sizes of the burn-in period and examine them by calculating the percentage of the convergence after each batch (we use batches of 1000 iterations). We finish the sampling process if at least 99.5% of the iterations have already converged and the sampling has reached a selected minimal number of iterations.

### 2.11 Inferring the variables based on the samples

For the real data, we compute final values of the inferred variables using multiple chains. Specifically, we run the model 20 times, each time with 10 chains. Then, from each run we select the chain with the highest likelihood, and all chains that obtained similar results. We consider two chains similar if the Pearson correlation between the inferred $H_{sk}$ values is higher than 0.9. If the chain with the highest likelihood does not have any other similar chains, we omit it. Afterward, we take an average out of all the chains that are similar to the one with the highest likelihood out of the remaining chains. The estimates from the individual runs were then combined using the two-stage consensus procedure described in the Supplementary Material (see Methods and Material; Supplementary Figs. S1-S4).

### 2.12 Differential gene expression analysis between clones

We obtain posterior pseudo-samples of the inferred gene expression per clone *k* and gene *g* using batch means computed from the post-convergence MCMC iterations. Specifically, the post-burn-in iterations are divided into batches of *b* MCMC steps; within each batch the b/t thinned $B_{kg}$ values (thinning factor *t*) are averaged to form one batch-mean pseudo-sample vector per chain. These batch-mean pseudo-samples are pooled across all consensus-selected chains and runs, yielding a set of *M* posterior pseudo-samples in total.

For each gene *g* and each pair of clones $\left(k_{1},k_{2}\right)$, we assess differential expression on the log2 fold-change scale using the Highest Density Interval plus Region of Practical Equivalence (HDI+ROPE) criterion (Kruschke 2018). To regularise fold-changes for genes with very low inferred expression, a pseudocount $\varepsilon_{0}$, set to one tenth of the median of the positive posterior-mean $B_{kg}$ values, is added before the log transform. Let $\delta_{g}={\;\text{log}\;}_{2}(B_{k_{1},g}+\varepsilon_{0})-{\;\text{log}\;}_{2}(B_{k_{2},g}+\varepsilon_{0})$ denote the posterior samples of the pairwise log2 fold-change in clone-specific expression. We compute the 95% HDI of $\delta_{g}$, defined as the shortest interval that contains 95% of the posterior mass. We define a Region of Practical Equivalence (ROPE) around zero, [−ε,+ε], where ε is set to the 10th percentile of the absolute log2 fold-changes $\left|{\;\text{log}\;}_{2}(B_{k_{1},g}+\varepsilon_{0})-{\;\text{log}\;}_{2}(B_{k_{2},g}+\varepsilon_{0})\right|$ evaluated at the posterior mean across all gene-clonepair combinations. This threshold defines the smallest fold-change that we treat as practically non-negligible in the context of the observed data. A gene *g* is declared differentially expressed between clones $k_{1}$ and $k_{2}$ if the 95% HDI of $\delta_{g}$ lies entirely outside the ROPE: either ${\text{HDI}}_{\text{low}}>\varepsilon$ (higher in $k_{1}$) or ${\text{HDI}}_{\text{high}}<-\varepsilon$ (higher in $k_{2}$). This criterion simultaneously requires that the fold-change is credibly non-zero and of practical magnitude, avoiding detection of statistically certain but biologically negligible differences.

To further characterise clone-specific transcriptional programmes, we performed Gene Set Enrichment Analysis (GSEA) for each clone *k* (excluding the normal clone). Genes were ranked by the posterior mean effect size relative to all other non-normal clones:


(2) $$\begin{array}{c} {\text{score}}_{k}(g)=E\left[{\;\text{log}\;}_{2}(B_{k,g}+\varepsilon_{0})|\text{Data}\right]\\ -\frac{1}{\left|K^{\prime }\right|}\sum_{k^{\prime }\in K^{\prime }}E\left[{\;\text{log}\;}_{2}(B_{k^{\prime },g}+\varepsilon_{0})|\text{Data}\right] \end{array}$$

where $K^{\prime }$ denotes the set of non-normal clones excluding *k*, $\varepsilon_{0}$ is the pseudocount defined above, and expectations are estimated as means over the pooled posterior pseudo-samples. This per-clone mean log2 fold-change is the standard input for pre-ranked GSEA.

### 2.13 Computing the expected gene expression values across spots based on the inferred clonal gene expression profiles

Having the inferred clonal expression $B_{kg}$, we compute the expected gene expression profile across spots in the tissue section *j* as:


$${\hat{Y}}_{sg}=\sum_{k}t_{j}N_{s}H_{sk}B_{kg}=t_{j}N_{s}\sum_{k}H_{sk}B_{kg}.$$

### 2.14 Generating simulated data

The data were simulated from the ClonalGE model. To this end, we first fixed the hyperparameters, and next sampled each variable conditional on its parents’ values, following the order of the variables in the graphical model (Fig. 1d).

Specifically, we generated data in three setups. First, the *normal* setup, with K=5 clones randomly mixed in S=300 spots, total number of mutations set to I=200 and the number of analyzed genes G=50. We fixed the hyperparameter $\Lambda_{s}$ so that the expected value for the number of cells was 45, and the hyperparameter $\zeta_{s}$ so that the expected number of clones in each spot *s* was 2.5. We fixed the hyperparameters *r* and *q* to 0.005 and 1 so that the sum of simulated read counts per each spot was in the (36, 44) interval, and the expected value of gene expression variance for the genes across the spots was 1715, reflecting the levels observed in real data.

Next, to test the influence of the different gene expression levels and the coverage per mutation, we designed two additional setups (*low expression variance* and *high coverage*). These setups were obtained as the *normal* setup, but lowering the expected value of the expression variance and increasing the average number of reads, respectively (see Table 1). For each setup, we repeated the simulation 20 times, obtaining a total of 60 simulated datasets.

**Table 1:** Parameters for generating three different simulation setups.

Setup			
Property	Normal		
Variance	Low expression		
Coverage	High		
Interval of the			
Average read counts	(36, 44)	(36, 44)	(180, 221)
Expected value of			
Expression variance	1715	315	1715

## 3 Results

### 3.1 ClonalGE outperforms predecessor model on simulated data

We first assessed the performance of ClonalGE on simulated data, where the ground truth was known, for three different simulation setups: *normal*, *low expression variance*, and *high coverage* (see Methods).

For inference, we first ran the sampling process of ClonalGE for at least 10 000 iterations, saving every 5th sample and stopping after reaching convergence with an upper bound of 30 000 iterations. The initial parameters were set using a pre-run of Tumoroscope (see Methods). We discarded the burn-in samples and used the remaining for inference of the latent variables. We used known values of the hyper-parameters, except for *r* and *q*, which were estimated (see Methods). We also evaluated another running procedure of ClonalGE that aimed at decreasing the chances of getting stuck in local optima. To this end, we ran five MCMC chains for ClonalGE and selected the chain with the highest likelihood. This procedure is referred to as ClonalGE 5 chains.

For comparison, we ran Tumoroscope with its parameter estimation procedure for the same lower and upper bound numbers of iterations as ClonalGE. To estimate gene expression per clone after running Tumoroscope, similarly to Shafighi *et al.* (2024), we used linear regression (LR) with weights fixed to the fractions of clones in spots inferred by Tumoroscope. Hence, this entire procedure is referred to as Tumoroscope + LR.

Overall, both ClonalGE and ClonalGE 5 chains obtained significantly better results compared to Tumoroscope + LR in all simulation setups (Fig. 2). In particular, ClonalGE obtained much more accurate estimates of gene expression values in clones (Fig. 2d). This result confirms that the joint modeling of the gene expression values with the mutation read counts as performed in ClonalGE gives increased statistical strength and yields higher performance than the double step procedure employed by combining Tumoroscope with linear regression. This advantage may also come from the appropriate model of the Negative Binomial distribution for the expression data per gene and spot employed by ClonalGE, in contrast to linear regression.

**Figure 2:**
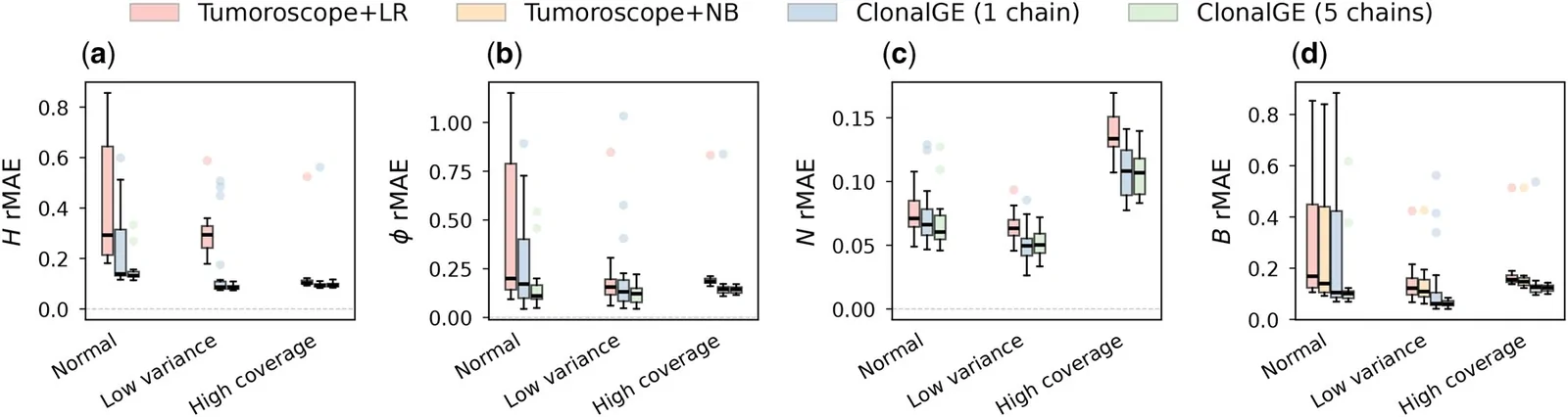
Validation on simulated data. Relative mean absolute error (rMAE) of inferred model variables across 20 independent simulation replicates under three conditions (Normal, Low variance, High coverage). Each panel shows rMAE for one variable: (a) clonal composition *H*, (b) per-cell read coverage ϕ, (c) cell count *N*, and (d) clone-specific gene expression *B*. Four methods are compared: Tumoroscope with linear-regression post-processing (Tumoroscope+LR), Tumoroscope with Negative Binomial regression post-processing (Tumoroscope+NB), ClonalGE run as a single Markov chain, and ClonalGE run as five independent chains with the best chain selected by log-likelihood. Tumoroscope+NB is only evaluated on *B* (panels a–c are identical to Tumoroscope+LR). Boxes show the interquartile range; horizontal lines indicate the median; whiskers extend to 1.5 IQR.

To disentangle the contribution of the Negative Binomial distribution from the benefit of joint inference, we additionally compared ClonalGE against Tumoroscope followed by Negative Binomial regression (Tumoroscope + NB), where gene expression per clone is estimated using a Negative Binomial generalized linear model with weights fixed to the Tumoroscope-inferred clone fractions. While Tumoroscope + NB improves upon Tumoroscope + LR for gene expression estimation (Fig. 2d, Supplementary Table S2), ClonalGE still achieves superior accuracy across all variables. This confirms that the integrated probabilistic inference framework provides benefits beyond adopting a more appropriate count distribution in a post-processing step (Fig. 2).

Interestingly, ClonalGE also outperforms Tumoroscope in the functionality that is shared between these two models: inference of the fraction of clones in spots (Fig. 2a), numbers of cells in spots (Fig. 2c), and read coverages per mutation per cell (Fig. 2b). This is likely due to the fact that ClonalGE, in contrast to Tumoroscope, benefits from the additional signal in gene expression measurements across spots. The most significant improvement over Tumoroscope is visible for the *normal* and *low expression variance* setups, both characterized by low coverage. These demanding setups illustrate that Tumoroscope is more sensitive to coverage than ClonalGE.

As expected, ClonalGE with five chains achieved more robust results with lower outliers compared to both ClonalGE and Tumoroscope + LR. The mean and median of the error in all the setups and variables decreased or stayed equal when we used five chains instead of one. These results emphasize the importance of running more chains to decrease the probability of the model getting trapped in the local minimum. ClonalGE also remained robust under simulations that violate its modelling assumptions, including zero-inflated gene expression and section-level batch effects (Supplementary Fig. S9).

### 3.2 ClonalGE reconstructs the gene expression profile of the clones on prostate cancer data

To showcase the performance of ClonalGE on real data, we applied it to prostate cancer dataset generated by Berglund *et al.* (2018). To this end, we selected 3 sections: SP1, SP2, and SP3 out of 12 sections, based on a pathologist’s annotation of cancer regions. These sections were sampled from one patient, for which deep DNA-seq and ST data (custom arrays) of neighboring layers were generated (Berglund *et al.* 2018). From these sections, only the spots overlapping with cancer regions were selected, namely 198, 70, and 43 spots from sections SP1, SP2, and SP3 respectively, out of 968–1001 spots per section. Furthermore, we excluded 17 spots in section SP1 that lacked gene expression data. In the remaining 294 spots, the sequenced transcripts were mapped to 12 873 different genes. We selected the data for the 1000 most variable genes to (i) limit the computation time and (ii) concentrate on the genes that carry the most information for more accurate inference. We note that this pre-selection strategy, while computationally efficient and focused on informative signals, may miss genes with subtle but consistent clone-specific expression patterns that do not exhibit high overall variance across spots. We used the mutations present in both DNA-seq and ST data together with the evolutionary tree reconstructed from DNA-seq data [Methods; Shafighi *et al.* (2024)]. The tree included a normal clone without somatic mutations and three other clones. Finally, we applied ClonalGE to deconvolute the transcriptomic signal from the 294 cancerous spots in the ST data in order to achieve the proportions of the underlying clones and the expression profile of the clones.

For running the model on the prostate cancer dataset, we used the same procedure and number of iterations as described for the simulated data. We repeated the sampling and inference 20 times, with different random seeds, and combined the 8 of the 20 runs that formed the consensus after clone alignment (see Supplementary Methods and Material; Figs. S1, S2, S3, and, S4). For the real data, the true values of the hyperparameters are unknown and estimated (see Methods).

For visualisation we selected the 30 genes with the highest variance of their log2 clone-specific expression, ${\text{Var}}_{k}({\;\text{log}\;}_{2}(B_{kg}+\varepsilon_{0}))$, i.e. the genes whose expression differs most across clones on the relative scale, and plotted their log_2_ expression across clones (Fig. 3a). The credibility of each clone-pair difference is shown separately by the HDI+ROPE test in Fig. 3b.

**Figure 3:**
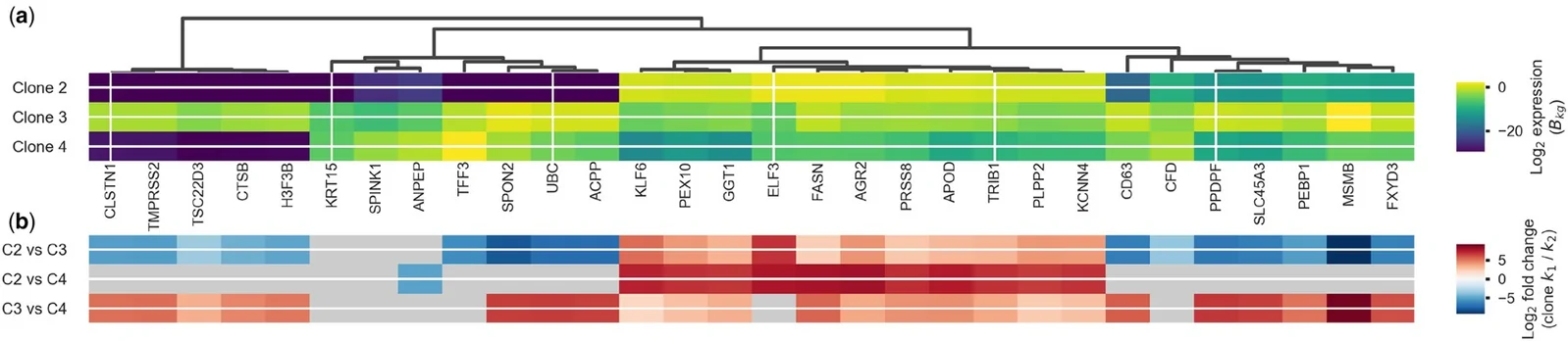
(a) Log_2_-transformed posterior mean clone-specific expression (${\;\text{log}\;}_{2}B_{kg}$) of the 30 genes with the highest variance of log2 clone-specific expression across clones (see Methods), for clones 2–4 in the prostate cancer sample. Genes are ordered by hierarchical clustering. (b) Posterior mean log2 fold-change (${\;\text{log}\;}_{2}(B_{k_{1},g}/B_{k_{2},g})$) for each clone pair and gene. Only genes declared differentially expressed by the HDI+ROPE criterion (on the log2 fold-change scale) are coloured (red: higher in $k_{1}$; blue: higher in $k_{2}$); grey cells indicate no credible practical difference (95% HDI overlaps the ROPE).

The selected genes are strongly enriched for prostate-epithelial and prostate-cancer-associated genes. Several are canonical prostate markers according to the Human Protein Atlas (Pontén *et al.* 2008), including *MSMB* (β-microseminoprotein), *ACPP* (prostatic acid phosphatase), *SLC45A3* (prostein), *TMPRSS2*, *SPON2* and *TFF3*. The set further contains genes repeatedly implicated in prostate cancer: *TMPRSS2*, the androgen-regulated gene that forms the recurrent TMPRSS2–ERG gene fusion; *SPINK1*, which marks an ETS-fusion-negative molecular subtype; *FASN* (fatty-acid synthase), a hallmark of the lipogenic phenotype of prostate cancer; together with *AGR2*, *PRSS8* (prostasin), *ELF3* and *KLF6*. Their differential expression across clones indicates that the inferred clones differ in their prostate luminal-secretory and oncogenic programmes. To further characterize clone-specific gene sets, we performed Gene Set Enrichment Analysis (GSEA) using the per-clone posterior mean log2 fold-change ranking ${\text{score}}_{k}(g)$ defined in Methods (Supplementary Fig. S8); clone 3 was enriched for the KEGG prostate-cancer pathway, consistent with its high expression of *MSMB*, *ACPP* and *SLC45A3*.

### 3.3 MCMC samples from ClonalGE enable differential gene expression analysis between clones

We applied the HDI+ROPE criterion to assess differential expression between each pair of clones directly from the MCMC samples (Methods; Fig. 3b). The majority of the top-ranked genes show credible and practically meaningful differential expression in at least one clone pair. Clone 3 was the most transcriptionally distinct clone, differing from clones 2 and 4 in the largest numbers of genes (530 and 630 of the 1000 analysed genes, respectively) and showing the highest expression of the prostate luminal-secretory markers *MSMB*, *ACPP*, and *SLC45A3* (Fig. 3a). By contrast, the greatest similarity was observed between clones 2 and 4, which differed in the fewest genes (138) and whose log_2_ expression profiles were the most correlated (Fig. 3a).

Spatially, clone 3 was the dominant clone across all three sections (mean inferred proportion 0.66; Fig. 4), consistent with its distinct luminal-secretory profile, whereas the minor clones were concentrated in specific sections, with clone 2 mainly in section SP1 and clone 4 mainly in section SP2. Interestingly, the distribution of the clones across the analyzed tissue sections inferred by ClonalGE is different than the one inferred by Tumoroscope [compare Fig. 5 in Shafighi *et al.* (2024)], which can be attributed to the adoption of the extra information of gene expression in the inference by ClonalGE.

**Figure 4:**
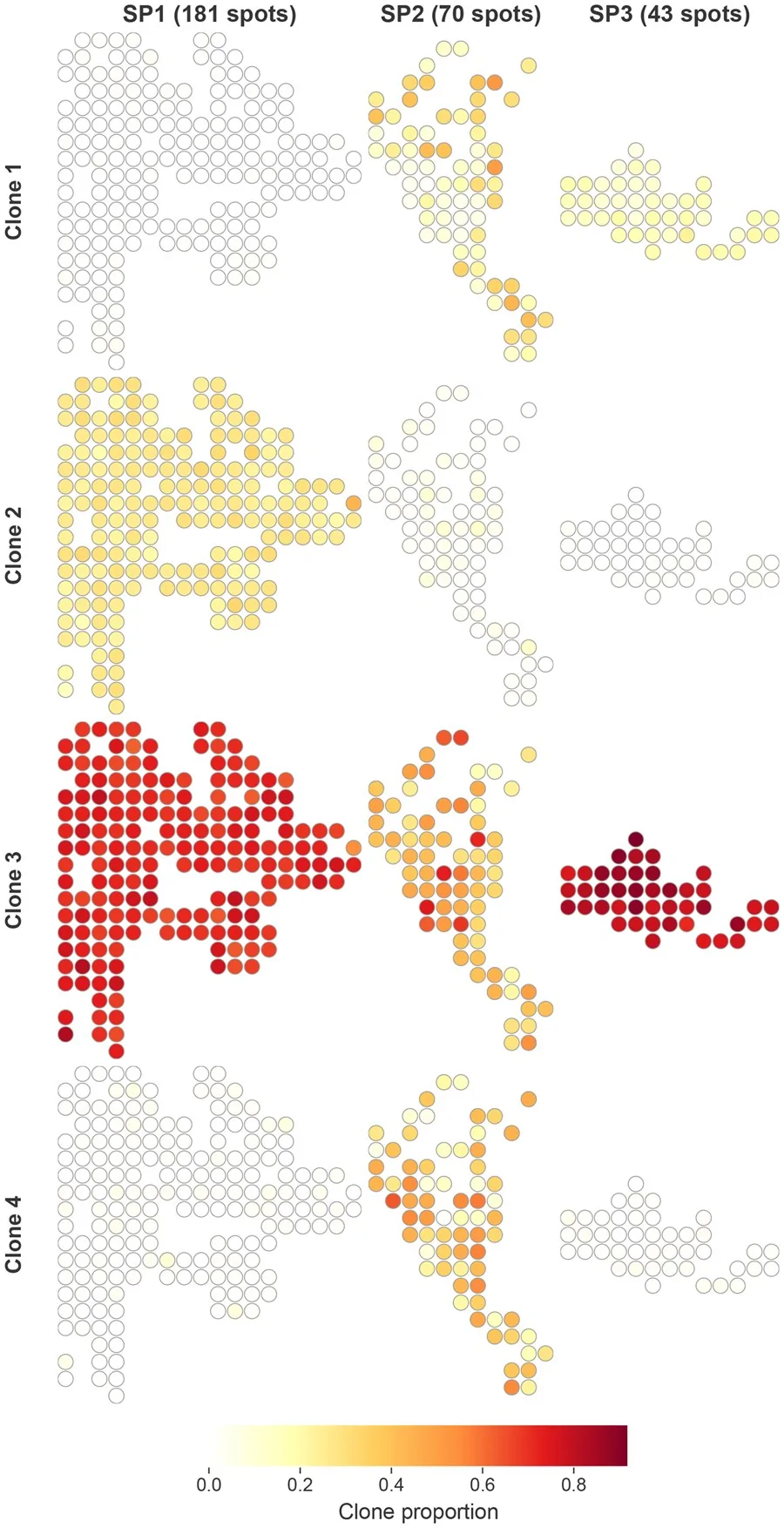
Spatial maps of inferred clonal composition. Each panel shows one clone (rows: Clone 1–4) and one tissue section (columns: SP1, SP2, SP3); spots are drawn as circles at their physical coordinates, coloured by the ClonalGE-inferred proportion of that clone in the spot (white = 0, dark red = high).

**Figure 5:**
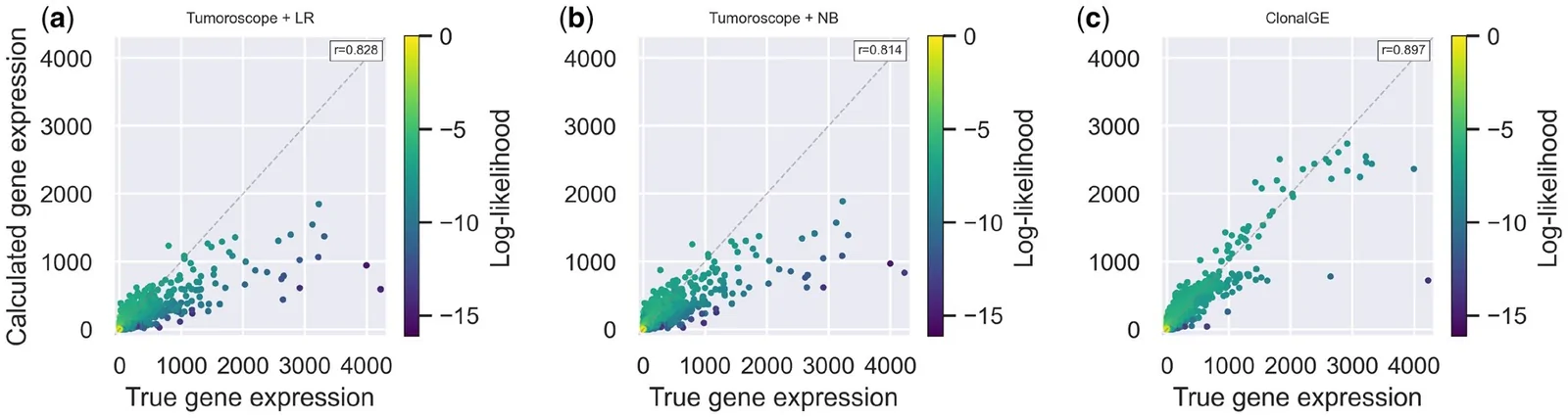
ClonalGE reconstructs spot-level gene expression more accurately than post-processing approaches applied to Tumoroscope. Scatter plots comparing measured (x-axis) versus model-predicted (y-axis) gene expression across all spots and genes in the prostate cancer sample, for three methods: (a) Tumoroscope followed by linear regression (Tumoroscope + LR; Pearson r=0.828), (b) Tumoroscope followed by Negative Binomial regression (Tumoroscope + NB; Pearson r=0.814), and (c) ClonalGE (Pearson r=0.897). Each point represents one gene–spot observation; colour encodes the per-observation log-likelihood under a Negative Binomial model (shared colour scale across panels, with darker colours indicating lower log-likelihood). ClonalGE achieves the highest correlation with the observed data, demonstrating that joint probabilistic modelling of gene expression and mutation read counts yields more accurate reconstruction of spatial expression patterns than the two-step approach of first running Tumoroscope and then fitting expression via regression. Results shown use the consensus of 8 out of 20 independent runs (approach 2; see Methods).

### 3.4 ClonalGE reconstructs the gene expression profile of the tissue more accurately than tumoroscope

Finally, we evaluated the accuracy of the gene expression reconstruction using ClonalGE. To this end, we compared the observed gene expression values of the spots measured in the prostate tissue with the values expected for the same genes at the same spots based on the model estimation of clone-specific gene expression profiles (the real $Y_{sg}$ values with the ${\hat{Y}}_{sg}$ values, see Methods for their calculation). For an accurate model, the real values should perfectly agree with the expected ones from the model.

For a comparison, we applied the entire procedure of Tumoroscope + LR and Tumoroscope + NB on the same data, with the same number of iterations as for ClonalGE, and also obtained the expected gene expression values at the spots to compare them to the real ones.

We observed a substantially better agreement between the observed gene expression values across spots and the model-predicted values for ClonalGE than for either comparison method (Pearson r=0.897, r=0.828, and r=0.814 for ClonalGE, Tumoroscope + LR, and Tumoroscope + NB respectively; Fig. 5). Both comparison methods show high agreement for small expression values. However, in contrast to ClonalGE, Tumoroscope + LR and Tumoroscope + NB tend to underestimate gene expression at high expression levels (Fig. 5a). Interestingly, the worst agreement is obtained for genes that also have the lowest log-likelihood, suggesting that Tumoroscope + LR fails to capture the count-data structure of gene expression. While Tumoroscope + NB introduces a Negative Binomial model in the post-processing step, it still lags behind ClonalGE, confirming that the benefit of the NB distribution is fully realised only when integrated jointly with the inference of clonal composition (Fig. 5b).With only a few exceptions, ClonalGE-predicted values closely match the observed expression values even at high expression levels (Fig. 5c).

This comparison reflects relative model fit, as all methods condition on the observed expression $Y_{sg}$ during inference. Nevertheless, the different levels of agreement remain informative, as they indicate how well each model captures the structure of the spatial expression signal. The slightly higher Pearson *r* for Tumoroscope + LR than for Tumoroscope + NB is consistent with the fact that linear regression directly optimizes squared reconstruction error, whereas NB post-processing instead targets a regularized count-data likelihood. By contrast, on simulated data with known ground-truth $B_{kg}$, Tumoroscope + NB achieves lower rMAE on *B* than Tumoroscope + LR (Fig. 2a), indicating more accurate recovery of clone-specific expression profiles.

### 3.5 Comparison with a reference-free expression deconvolution baseline

To address the question of whether purely expression-based deconvolution methods can recover clonal structure, we benchmarked ClonalGE against STdeconvolve (Miller *et al.* 2022), the closest methodologically appropriate baseline among reference-free approaches. STdeconvolve applies Latent Dirichlet Allocation (LDA) to spatial gene expression counts without requiring any external reference, and outputs per-spot topic proportions ($\hat{\theta }$) and per-topic gene expression profiles ($\hat{\beta }$), which are structurally analogous to ClonalGE’s inferred clone proportions *H* and clone-specific expression matrix *B*, respectively.

Critically, unlike the methods commonly used for cell-type deconvolution in spatial transcriptomics [e.g. RCTD (Cable *et al.* 2022), Cell2location (Kleshchevnikov *et al.* 2022), SPOTlight (Elosua-Bayes *et al.* 2021), Stereoscope (Andersson *et al.* 2020), STRIDE (Sun *et al.* 2022)], STdeconvolve requires no paired single-cell RNA-sequencing reference, making it the only existing method that can be applied under comparable data conditions to ClonalGE. Furthermore, none of the reference-based deconvolution methods model somatic mutations or clonal identity; their decomposition targets spot-level cell-type mixtures rather than the tumour clone structure inferred by ClonalGE, which precludes a direct comparison.

We evaluated both methods on 20 independent simulation replicates across three simulation configurations that vary in expression variance and sequencing depth (Normal, Low Variance, High Coverage; see Methods). In all cases, the ground truth clone composition matrix $H^{\text{true}}$ (spots × clones) and clone-specific expression matrix $B^{\text{true}}$ (clones × genes) were known. Since STdeconvolve’s LDA topics are not inherently labelled, we resolved the clone–topic correspondence prior to evaluation using the Hungarian algorithm (Kuhn 1955), which finds the permutation of predicted topics that minimises the total MAE for *H* relative to the ground truth, i.e. the most favourable possible assignment for STdeconvolve. To enable a scale-free comparison of *B*, both the ground truth $B^{\text{true}}$ and the inferred matrices from both methods were row-normalised to unit sum, representing the relative gene expression profile per clone. Recovery accuracy was quantified using the Mean Absolute Error (MAE) for both *H* and *B*.

ClonalGE achieved substantially lower MAE for *H* than STdeconvolve across all three configurations (Fig. 6; Supplementary Table ??), 0.0301 vs. 0.0682 under the Normal setting (2.3-fold improvement), 0.0174 vs. 0.0463 under Low Variance (2.7-fold), and 0.0191 vs. 0.0683 under High Coverage (3.6-fold). The advantage was even more pronounced for clone-specific expression recovery (The MAE for *B*), ClonalGE achieved 3.0-, 5.2-, and 3.2-fold lower error in the Normal, Low Variance, and High Coverage settings, respectively (Supplementary Table S4). The consistently larger improvement in the High Coverage setting is particularly informative: deeper sequencing amplifies the information content of the allele-frequency signal, which ClonalGE leverages through its mutation likelihood term but STdeconvolve cannot exploit. These results confirm that integrating somatic mutation data is not merely complementary to expression-based deconvolution, but is essential for accurately recovering tumour clonal composition in spatial transcriptomics data. An expression-only approach, even under the most favourable conditions (known *K*, optimal topic matching), recovers clone proportions with 2–4-fold higher error than ClonalGE, and fails to approach the accuracy achievable when the mutation genotype of each clone is incorporated.

**Figure 6:**
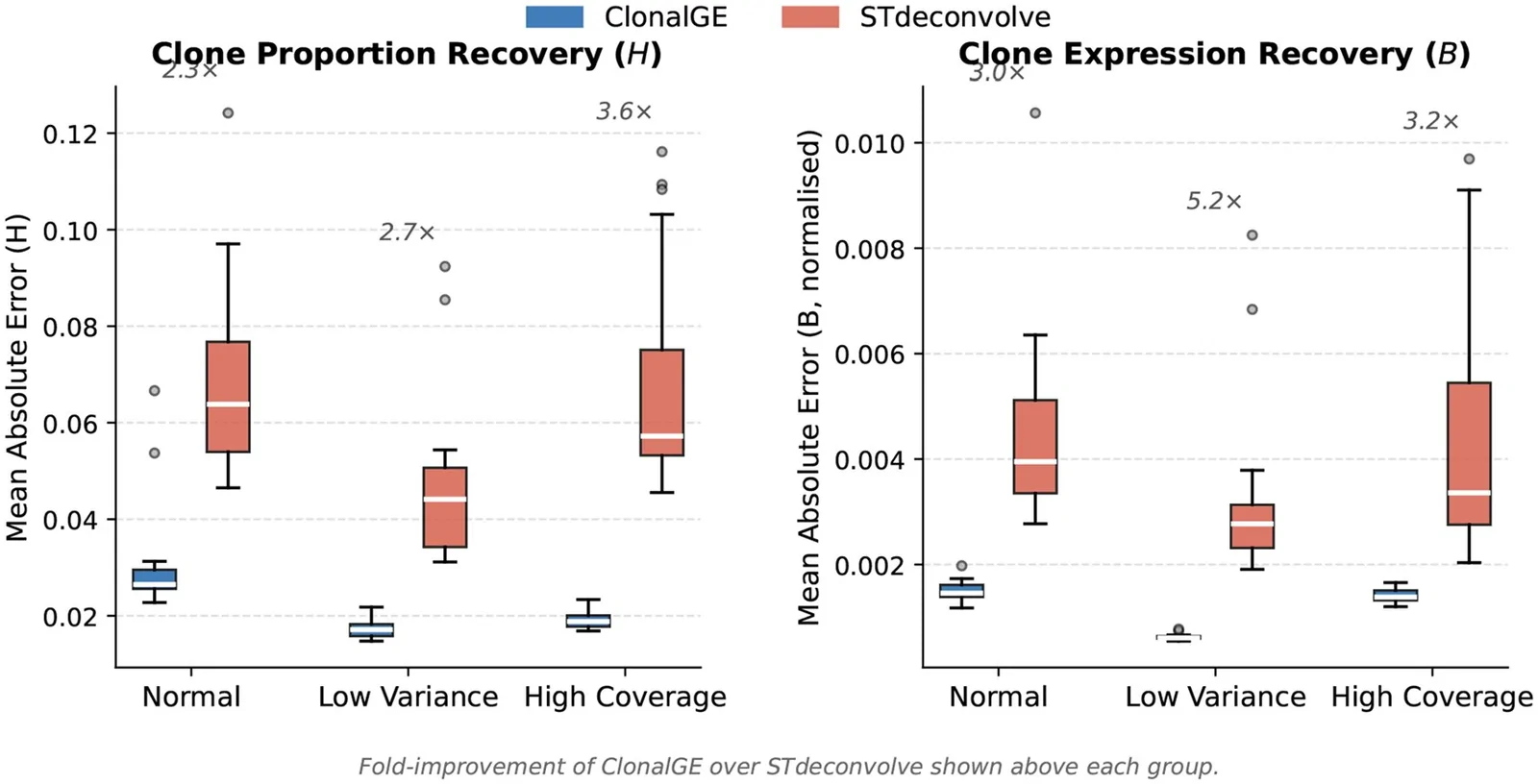
ClonalGE recovers clonal composition and clone-specific gene expression more accurately than the reference-free baseline STdeconvolve across all simulation settings. Boxplots show the Mean Absolute Error (MAE) over 20 independent simulation replicates for clone proportion estimation (*H*, left) and normalised clone-specific gene expression (*B*, right) under three simulation configurations: Normal, Low Variance, and High Coverage. Fold-improvements of ClonalGE over STdeconvolve are annotated above each configuration group. Lower values indicate better recovery of the ground truth.

## 4 Discussion

This study demonstrates that by a probabilistic approach to the integration of ST, DNA-seq and H&E image data, we can robustly identify the tumor clones, their specific gene expression profiles and map the spatial distribution of these clones within a tissue. Moreover, the inferred gene expression profile distribution of the clones enables us to perform differential expression analysis based only on a single biological sample from the tumor.

The core of the ClonalGE framework is a probabilistic graphical model that extends a previous model, Tumoroscope, by incorporating additional variables for gene expression in the spots. The model assumes that each clone has a specific average gene expression profile and that the expression of genes observed at the spots is a mixture of the gene expressions in the clones present in those spots. Overall, this framework provides a comprehensive approach for inferring and analyzing gene expression profiles of cancer clones within a tissue.

We note that the assumption of a clone-specific average gene expression profile that is constant across spatial locations is a simplification. In reality, the tumor microenvironment varies spatially (e.g. hypoxic regions, proximity to vasculature), and clone expression can be influenced by cell-cell interactions and paracrine signaling. Transcriptional plasticity may further allow cells to adapt to local conditions independently of their genotype. Our gene selection strategy, focusing on the most variable genes across tissue, aims to prioritize clone-specific signals over microenvironment-driven variation. Incorporating spatial covariates into the model or employing dedicated methods for identifying clone-specific genes are promising directions for future extensions.

We emphasize that ClonalGE addresses a fundamentally different problem from established spatial cell-type deconvolution methods such as RCTD (Cable *et al.* 2022), Cell2location (Kleshchevnikov *et al.* 2022), SPOTlight (Elosua-Bayes *et al.* 2021), Stereoscope (Andersson *et al.* 2020), and STRIDE (Sun *et al.* 2022). These tools decompose spot expression into contributions from known cell types, typically requiring a single-cell RNA-seq reference atlas. In contrast, ClonalGE performs clonal deconvolution, identifying cancer subclone compositions by integrating somatic SNV information from DNA sequencing with spatial transcriptomics. The clones are defined by their evolutionary (mutational) relationships rather than by transcriptomic cell-type signatures, and a direct comparison would require single-cell data with known clonal labels which is precisely the information that ClonalGE is designed to infer without such data.

The ClonalGE model was determined to be superior to the framework where the predecessor Tumoroscope is followed by linear regression (Tumoroscope + LR) in all simulated scenarios. ClonalGE was able to provide more precise estimates of gene expression levels in clones by incorporating information on mutation read counts. Additionally, by utilizing this extra data, the ClonalGE model was found to have a better performance in determining the fraction of clones in spots, the number of cells in spots, and read coverages per mutation per cell. We also found that running multiple chains improves the robustness of the results by reducing the chance of the model getting stuck in local optima.

On the one hand, since the population of tumor cells co-evolves in a relatively restricted area in the body, the subclones are not expected to show major differences in their expression profiles. On the other hand, the occurrence of mutations may cause changes in gene expression, some of which may lead to phenotypic changes in the cells that carry those mutations. Previous studies did not decisively determine whether the genotypes of the clones determine their gene expression. One previous analysis by Kinker *et al.* (2020), decomposing gene expression programs purely from scRNA-seq data, found only a little overlap of cell subpopulations expressing different functional programs with their genotypes. Another study by Oksza-Orzechowski *et al.* (2024), integrating whole exome and single cell RNA sequencing in follicular lymphoma, found that the links between genotypes of the clones and their transcriptional phenotypes have different, patient-specific strengths. While one sample demonstrated no genotype-phenotype relation, another revealed clonal pathogenic mutations that could trigger specific transcriptional phenotypes.

Here, using ClonalGE on prostate cancer data, we were able to detect genes commonly found mutated in prostate cancer, and simultaneously infer their clone-specific expression. Our study indicates that there exist some key genes with variable activation across the clones. Moreover, we revealed a possible link between the location of the clones and their gene expression profiles. These results suggest that ClonalGE has the potential to be a valuable tool for bridging genomic and spatial heterogeneity with transcriptional plasticity, increasing our understanding of the genetic and transcriptional heterogeneity of cancer and informing the development of targeted treatments.

The clone-specific expression patterns inferred by ClonalGE in the prostate cancer sample suggest several testable hypotheses. The clones differ markedly in canonical prostate luminal-secretory genes: *MSMB*, *ACPP* (prostatic acid phosphatase) and *SLC45A3* (prostein) are highest in clone 3, identifying it as the most differentiated luminal-secretory clone, whereas the other clones express these genes at lower levels. Clone-specific expression of *TMPRSS2* (the androgen-regulated partner of the recurrent TMPRSS2–ERG fusion), *SPINK1* (a marker of an ETS-fusion-negative subtype) and the lipogenic enzyme *FASN* points to clone-to-clone differences in androgen signalling and oncogenic programmes, and identifies candidates for spatial validation in larger cohorts. Together with the GSEA results, in which clone 3 was enriched for the KEGG prostate-cancer pathway, these findings illustrate how ClonalGE can generate clone-resolved hypotheses that are not accessible from bulk DNA-seq, bulk ST, or single-cell RNA-seq alone.

Clinically, the ability to resolve clone composition spatially within a tumour section opens several translational directions. First, identifying clones that express therapeutic targets (e.g. AR-pathway components or kinase signalling genes) at high levels (and locating them within the tissue) could inform combination therapies that account for intra-tumour heterogeneity rather than treating the tumour as a single transcriptional entity. Second, mapping clones to spatial regions with distinct microenvironmental features (e.g. proximity to stromal tissue, immune infiltrate, or vasculature) using H&E-paired ST cohorts could reveal which clonal phenotypes are favoured by which microenvironments, suggesting selection pressures that drive disease progression. Third, the framework can in principle be applied to pre- and post-treatment biopsies from the same patient to identify which clones survive therapy and what transcriptional programmes characterise the resistant subpopulations, providing direct insight into clonal evolution under treatment. While the present study focuses on a single prostate cancer sample as a proof of concept, these directions illustrate the breadth of biological and clinical questions that an integrated clonal-deconvolution framework can begin to address.

In our analysis, we assumed that not all, but a subset of genes may show clone-specific gene expression. Accordingly, we estimated expression profiles of 1000 genes that had the most variable expression across ST spots, presuming that these have the most potential to be clone-specific. Including non-specific genes in the analysis would result in the incorporation of noise and could negatively affect the results. As an idea for future extension of the model, we could incorporate an additional hidden variable indicating whether the gene is clone specific or not. This information could then be inferred from the data. The per-iteration computational complexity of ClonalGE scales as O(S×K×(I+G)), where *S* is the number of spots, *K* the number of clones, *I* the number of mutations, and *G* the number of genes. Runtime analysis and scalability experiments are provided in the Supplementary Materials (S5).

The resolution of clonal spatial mapping depends on the number of detectable somatic mutations shared between DNA-seq and ST data. Mutation sparsity may limit the ability to resolve fine-grained spatial patterns of clone distribution within tissue sections, which may explain the limited spatial structure observed for certain sections in the prostate cancer analysis. Future datasets with deeper sequencing and higher mutation counts could improve spatial resolution. Incorporating explicit spatial priors that account for the expected contiguity of clonal populations is a promising direction for future model development.

Already in its current form, the model yields important insights into clonal phenotypes, marker genes, and their localization, taking a step forward in the analysis of clonal gene expression across tumor tissues.

## Supplementary Material

btag717_Supplementary_Data

## Data Availability

The prostate cancer dataset analysed in this study was originally published by Berglund et al. (2018) and previously processed and analysed by Shafighi et al. (2024). The original sequencing data are deposited in the European Genome-phenome Archive under accession EGAS00001003001, with access subject to approval by the relevant Data Access Committee. Processed prostate cancer data are available through the Tumoroscope GitHub repository, as described by Shafighi et al. (2024). Benchmarking datasets and intermediate results for the present study are available on Zenodo at https://doi.org/10.5281/zenodo.20446991.
