# Supplementary material for: Inferring gene expression profiles of tumor clones
Source: Bioinformatics. 2026 Oct 3;42(10):btag717. doi: 10.1093/bioinformatics/btag717 (PMC13681668; doi:10.1093/bioinformatics/btag717)
Supplement: Supplementary file 1 [file btag717_supplementary_data.pdf]

Supplementary Material

Inferring gene expression profiles of tumor clones

Shadi Shafighi, Barbara Jurzysta, Ewa Szczurek

Supplementary Material

MCMC convergence and model identifiability

To address concerns about model identifiability and the reproducibility of inference under a complex posterior, we ran ClonalGE on the prostate cancer dataset with 20 independent runs, each comprising ten parallel MCMC chains initialised from different random seeds. The analysis proceeded in two stages.

Stage 1: Within-run chain selection.
Within each run, the chain with the highest log-likelihood was identified as the within-run reference. Every other chain was compared to the reference by computing the Pearson correlation between their flattened posterior mean clonal composition matrices H. Chains with $r > 0.9$ were retained; the remainder were discarded. The within-run posterior estimate was obtained by element-wise averaging of all retained chains' posterior mean arrays for H, B, and N.

Stage 2: Consensus cross-run selection with Hungarian clone alignment.
Because mixture models are invariant to relabelling of latent components, clone indices may differ across independent runs (label switching). For each candidate reference run, every other run's H was realigned to the candidate using the Hungarian algorithm: a $K \times K$ cost matrix of negative Pearson correlations between clone columns was minimised with a linear assignment solver, yielding the clone permutation that maximises inter-run agreement; the same permutation was applied to B. After alignment, the number of runs agreeing with the candidate reference ($r > 0.9$ on the flattened aligned H) was counted. The candidate maximising this count was designated the *consensus* run (ties broken by highest mean r among agreers). The final posterior estimates were the element-wise means of the aligned arrays from all runs in the consensus group. Results are presented in Supplementary Figures S1–S5.

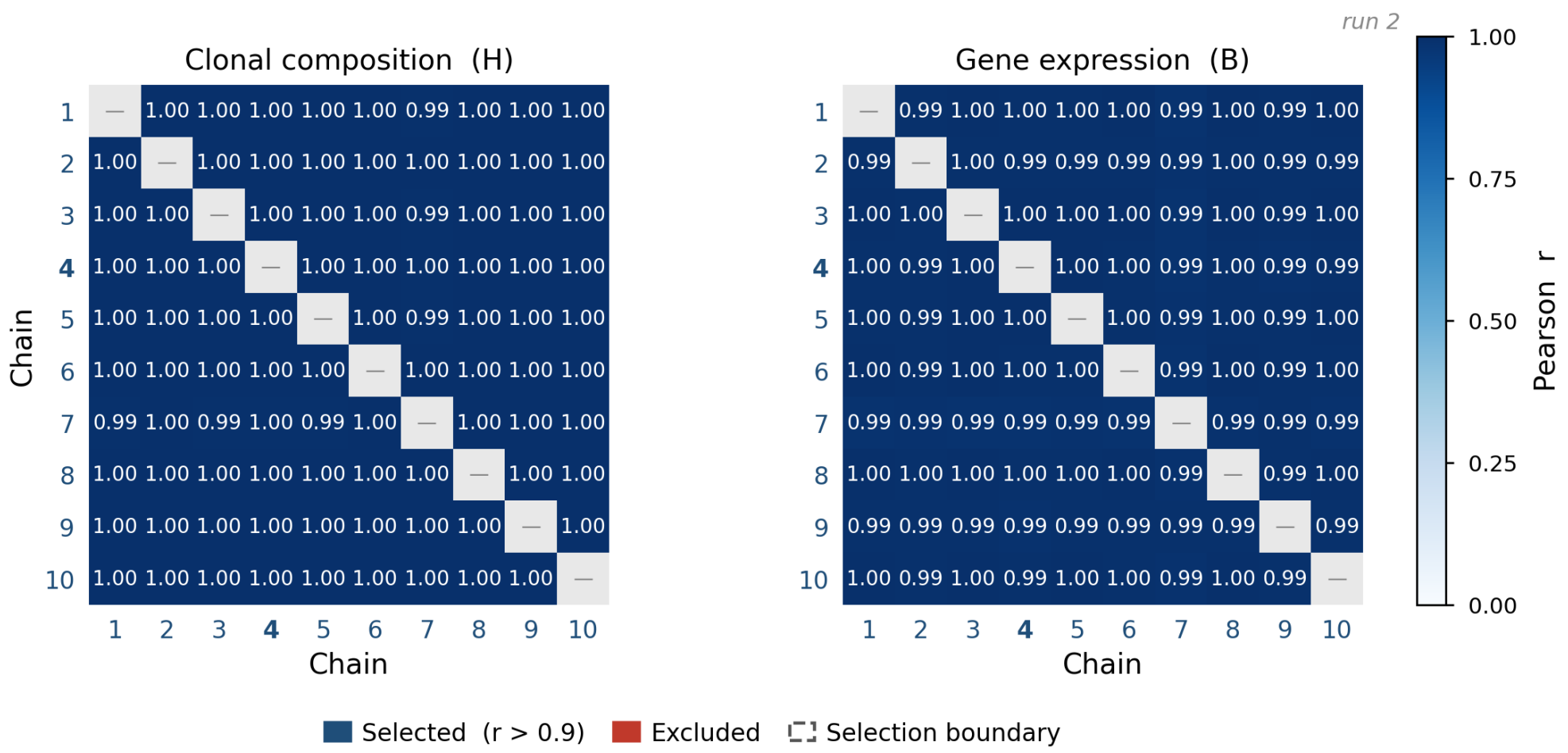

Fig. S1. Within-run MCMC chain agreement for the run with the highest log-likelihood (run 2). Pairwise Pearson correlation coefficients between the posterior mean estimates of all ten independent MCMC chains within the best-performing run, shown separately for the clonal composition matrix H (spots $\times$ clones; left) and the clone-specific gene expression matrix B (clones $\times$ genes; right). For each pair of chains (i, j), the Pearson correlation was computed between their flattened `inferred_H` (resp. `inferred_B`) arrays, giving two symmetric 10×10 matrices. Chain 4 (bold) achieved the highest log-likelihood within the run and served as the within-run reference (threshold $r > 0.9$); chains are reordered so that retained chains (blue labels) appear before the dashed selection boundary, with excluded chains (red labels) after it. All ten chains were retained (minimum pairwise $r = 0.99$ for both H and B), demonstrating that independently initialised Gibbs samplers converge to a consistent posterior solution within a single run.

Runtime analysis

We measured wall-clock runtimes across all simulation replicates and all real-data runs to characterise the computational demands of ClonalGE relative to its predecessor Tumoroscope. All experiments were executed on the same hardware (single workstation, CPU only), so runtimes are directly comparable across methods.

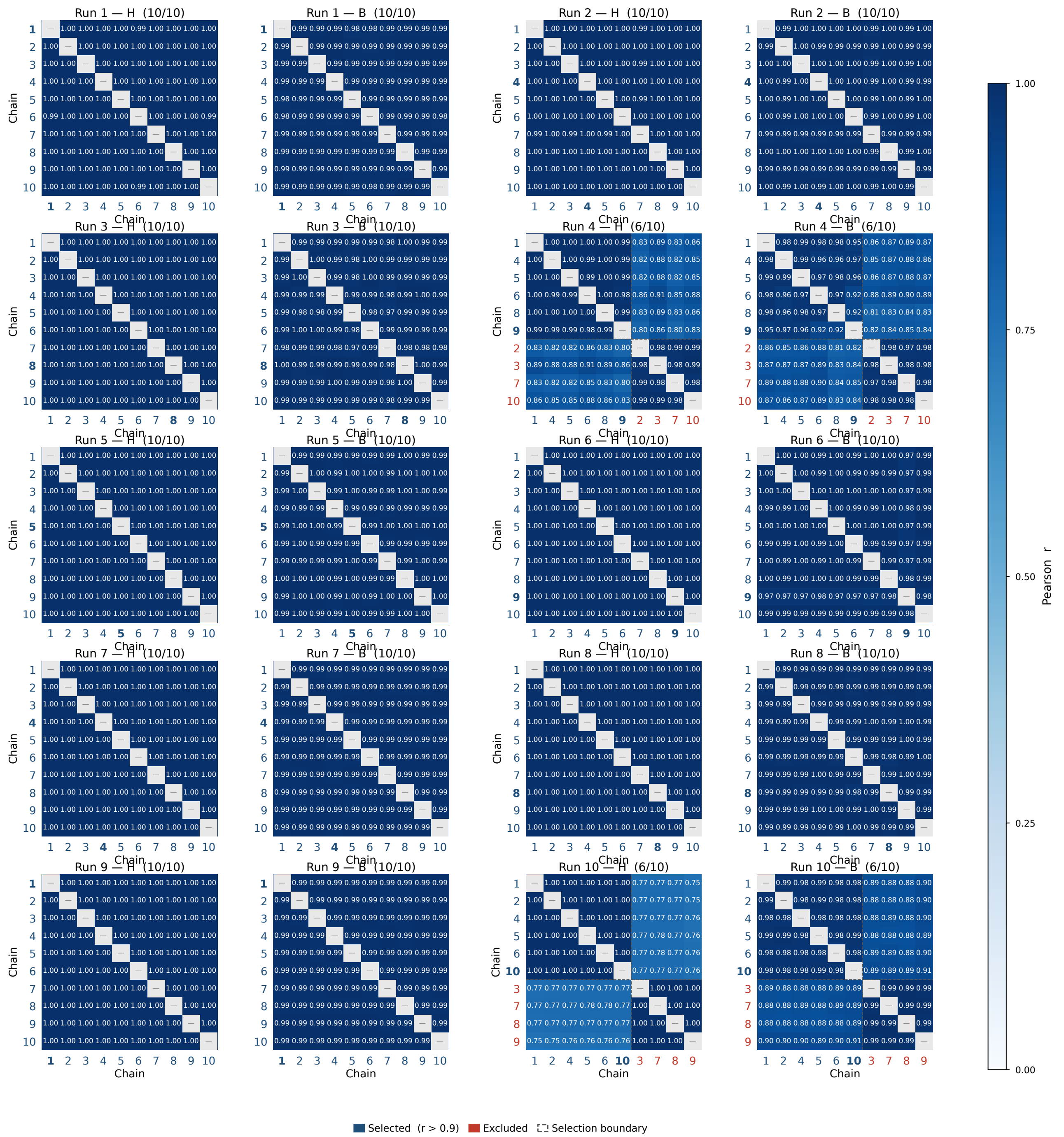

Fig. S2. Within-run MCMC chain agreement across all 20 independent runs. The same pairwise Pearson correlation procedure as in Supplementary Figure S1 was applied independently to each of the 20 runs. Within each panel the highest-loglik chain (bold) serves as the within-run reference; chains with $r > 0.9$ are labelled in blue and placed before the dashed selection boundary, while excluded chains are labelled in red. The panel title reports the number of retained chains. In all runs, retained chains show pairwise $r \geq 0.97$ for H and $r \geq 0.87$ for B, confirming robust within-run convergence. Here we show run 1 to 10.

Simulated data.

For each of the three simulation setups (*normal*, *low expression variance*, *high coverage*) we timed 20 independent replicates for each of the three methods: Tumoroscope, ClonalGE with a single MCMC chain (ClonalGE 1-chain), and ClonalGE with five parallel MCMC chains with best-chain selection (ClonalGE 5-chains). All methods used the same iteration bounds (minimum 10 000, maximum 30 000 iterations, saved every 5th sample) and stopped early upon satisfying the 99.5% Geweke convergence criterion. Summary statistics (mean ± standard deviation and range) are given in Table S5; runtime distributions are shown in Supplementary Figure S6.

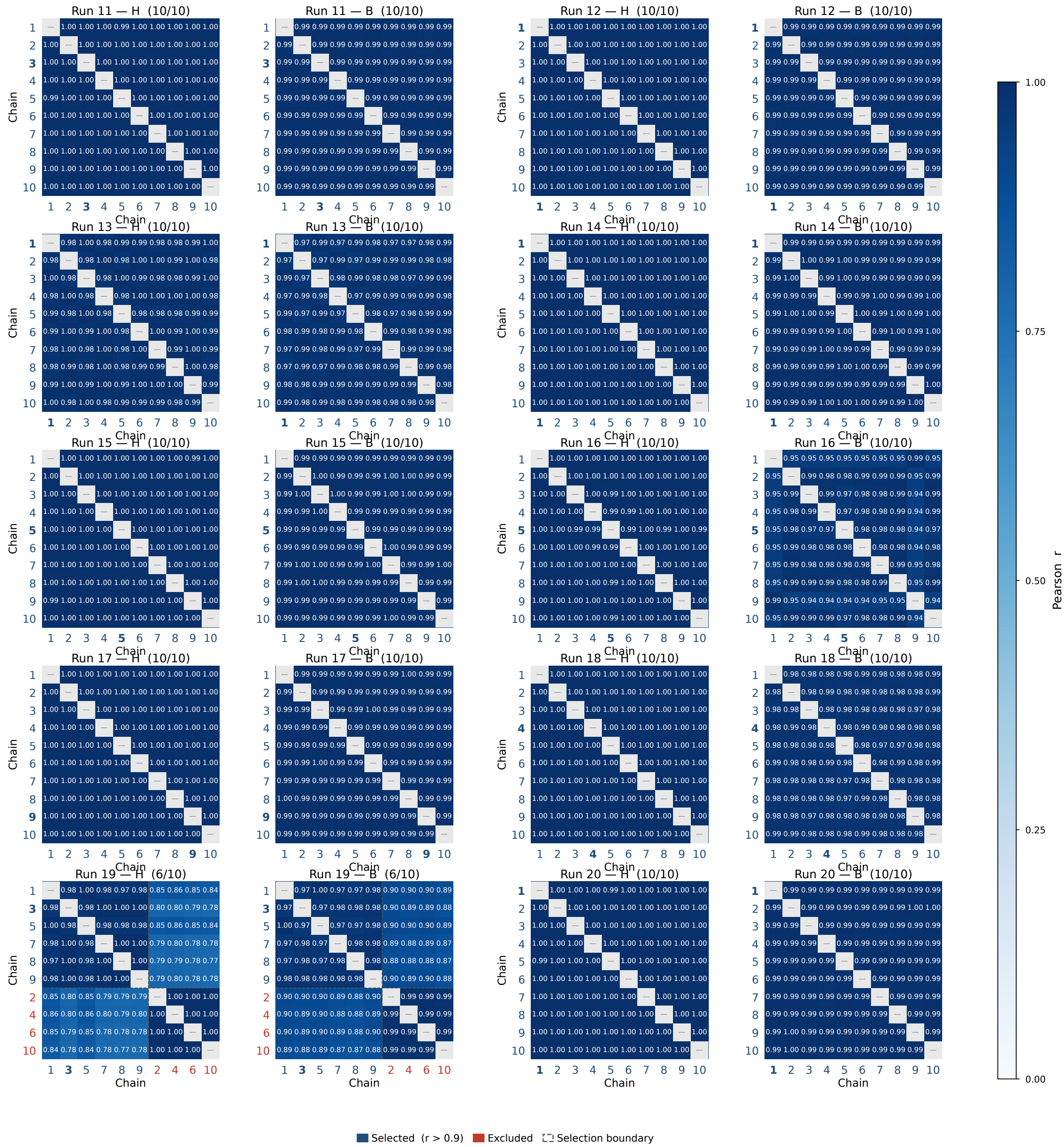

Fig. S3. Within-run MCMC chain agreement across all 20 independent runs. The same pairwise Pearson correlation procedure as in Supplementary Figure S1 was applied independently to each of the 20 runs. Within each panel the highest-loglik chain (bold) serves as the within-run reference; chains with $r > 0.9$ are labelled in blue and placed before the dashed selection boundary, while excluded chains are labelled in red. The panel title reports the number of retained chains. In all runs, retained chains show pairwise $r \geq 0.97$ for H and $r \geq 0.87$ for B, confirming robust within-run convergence. Here we show run 11 to 20.

Real prostate cancer data.

We ran ClonalGE on the prostate cancer dataset (294 spots, $K = 4$ clones, I mutations, $G = 1000$ genes) 20 times with different random seeds, each time executing 10 parallel MCMC chains. The mean wall-clock time per run was 769.8 ± 187.7 min (median 784.4 min, range 520.3–1047.4 min).

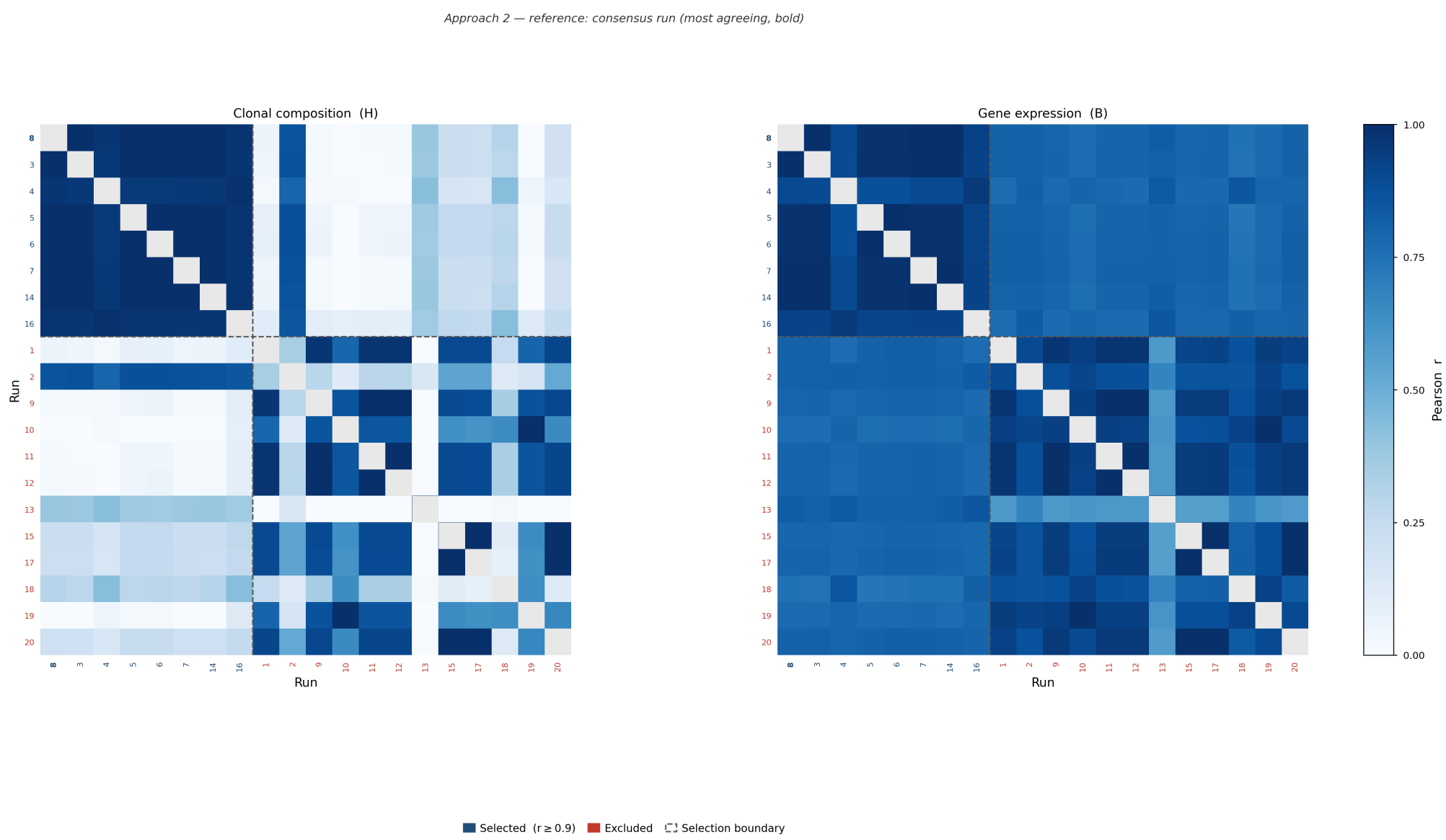

Fig. S4. Across-run agreement over 20 independent runs after consensus-based clone alignment. Each run's posterior estimate of H and B was obtained by within-run chain selection and averaging (Stage 1). Clone indices were then aligned across runs using the Hungarian algorithm (Stage 2). Run 8 (bold) was identified as the consensus, with eight of the 20 runs satisfying $r > 0.9$ on the aligned H (runs 3, 4, 5, 6, 7, 8, 14, 16; blue labels); the remaining 12 runs (red labels) were excluded. The 20×20 pairwise Pearson correlation matrices are shown for the aligned H (left) and B (right), with runs reordered so the consensus group appears first. Within the selected group, mean pairwise $r = 0.99$ (minimum 0.96) for H and mean $r = 0.95$ (minimum 0.88) for B, confirming reproducible recovery of clonal composition and gene expression structure. The excluded runs had converged to a different clone ordering that could not be reconciled after alignment, consistent with the known label-switching behaviour of Bayesian mixture models.

Table S1. Complete list of all variables and parameters in the ClonalGE model. Index ranges: $k \in \{1, \ldots, K\}$ (clones), $s \in \{1, \ldots, S\}$ (spots), $g \in \{1, \ldots, G\}$ (genes), $i \in \{1, \ldots, I\}$ (mutation loci), $j \in \{1, \ldots, J\}$ (tissue sections).

Symbol	Name	Dimension	Obs./Latent	Description
Indices and dimensions				
K	Number of clones	scalar	input	Number of clones inferred from DNA-seq
S	Number of spots	scalar	input	Number of cancerous ST spots
G	Number of genes	scalar	input	Number of genes analysed in ST data
I	Number of mutations	scalar	input	Number of somatic SNV loci shared between DNA-seq and ST
J	Number of sections	scalar	input	Number of tissue sections
Observed variables (given as input to the model)				
F_k	Clone frequency	K	observed	Frequency of clone k in the whole sample, estimated from DNA-seq
C_{ik}	Clone genotype matrix	$I \times K$	observed	Fraction of alleles of clone k carrying a mutation at locus i; values in $[0, 1]$
D_{is}	Total read depth	$I \times S$	observed	Total read count at mutation locus i in spot s from ST data
A_{is}	Alternated read count	$I \times S$	observed	Mutated (alternated) read count at locus i in spot s from ST data
Y_{sg}	Spot gene expression	$S \times G$	observed	UMI count of gene g in spot s from ST data
Λ_s	Expected cell count	S	observed	Cell count at spot s estimated from H&E images; used as Poisson rate for N_s
Latent variables (inferred by Gibbs/MH sampling)				
Π_{sk}	Clone presence prior	$S \times K$	latent	Beta-distributed probability that clone k is present in spot s
Z_{sk}	Clone presence indicator	$S \times K$	latent	Binary; $Z_{sk} = 1$ if clone k is present in spot s
G_{sk}	Unnorm. clone abundance	$S \times K$	latent	Gamma-distributed unnormalised abundance of clone k in spot s
H_{sk}	Clone fraction	$S \times K$	latent	Fraction of spot s occupied by clone k; $H_{sk} = G_{sk}/\sum_l G_{sl}$
Φ_{ik}	Per-cell read rate	$I \times K$	latent	Average read count at locus i per cell of clone k
N_s	Cell count	S	latent	Number of cells in spot s; Poisson-distributed with rate Λ_s
B_{kg}	Clonal gene expression	$K \times G$	latent	Average expression of gene g per cell in clone k; Gamma-distributed
Fixed hyperparameters and pre-estimated parameters				
ζ_s	Clone-count hyperparam.	S	hyperparameter	Controls expected number of clones per spot; shapes Beta prior on Π_{sk}
F_0	Absent-clone pseudo-count	scalar	hyperparameter	Gamma shape for G_{sk} when $Z_{sk} = 0$; set to a small positive value
r	Φ shape hyperparam.	scalar	hyperparameter	Shape parameter of Gamma prior on Φ_{ik}; estimated from data
q	Φ rate hyperparam.	scalar	hyperparameter	Rate parameter of Gamma prior on Φ_{ik}; estimated from data
α_g	B shape hyperparam.	G	hyperparameter	Per-gene shape parameter of Gamma prior on B_{kg}; estimated from data
β	B rate hyperparam.	scalar	hyperparameter	Rate parameter of Gamma prior on B_{kg}; estimated from data
p_g	NB success probability	G	hyperparameter	Success probability of Negative Binomial distribution for Y_{sg}; estimated from data
t_j	Section scaling factor	J	hyperparameter	Multiplicative scaling factor correcting for batch effects across section j
ω_B	B variance correction	scalar	hyperparameter	Correction constant for underestimation of β; fixed to 2
ω_ϕ	Φ variance correction	scalar	hyperparameter	Correction constant for underestimation of r; fixed to 2

Table S2. Median rMAE [IQR] for all inferred variables across 20 simulation replicates ($n = 20$ per condition). Bold: lowest median in each condition–metric group. Significance of ClonalGE improvement over Tumoroscope followed by linear regression (Tumoroscope+LR; one-sided Wilcoxon signed-rank test): $^{*}p < 0.05$, $^{**}p < 0.01$, $^{***}p < 0.001$. Tumoroscope followed by Negative Binomial regression (Tumoroscope+NB) is evaluated on B only; H, ϕ, N are identical to Tum+LR (—).

Condition	Method	H	ϕ	N	B
Normal	Tum+LR	0.293 [0.214, 0.644]	0.200 [0.143, 0.789]	0.071 [0.064, 0.085]	0.168 [0.124, 0.449]
	Tumoroscope+NB	—	—	—	0.140 [0.105, 0.440]
	ClonalGE (1-chain)	0.139 [0.132, 0.315]***	0.171 [0.099, 0.402]**	0.066 [0.058, 0.078]	0.105 [0.086, 0.423]*
	ClonalGE (5-chain)	**0.132 [0.128, 0.148]***	**0.111 [0.095, 0.166]***	**0.060 [0.055, 0.073]**	**0.101 [0.083, 0.111]***
Low variance	Tum+LR	0.294 [0.242, 0.329]	0.156 [0.117, 0.196]	0.063 [0.058, 0.070]	0.122 [0.096, 0.161]
	Tumoroscope+NB	—	—	—	0.109 [0.088, 0.155]
	ClonalGE (1-chain)	0.087 [0.079, 0.109]**	0.132 [0.084, 0.192]	**0.050 [0.042, 0.055]***	0.062 [0.056, 0.105]
	ClonalGE (5-chain)	**0.086 [0.079, 0.091]***	**0.122 [0.078, 0.150]***	0.050 [0.044, 0.059]***	**0.061 [0.054, 0.073]***
High coverage	Tum+LR	0.103 [0.100, 0.113]	0.185 [0.177, 0.200]	0.134 [0.127, 0.151]	0.155 [0.145, 0.173]
	Tumoroscope+NB	—	—	—	0.148 [0.133, 0.162]
	ClonalGE (1-chain)	**0.093 [0.088, 0.099]***	0.145 [0.126, 0.157]***	0.108 [0.089, 0.125]***	0.126 [0.106, 0.134]***
	ClonalGE (5-chain)	0.094 [0.089, 0.100]***	**0.144 [0.126, 0.156]***	**0.107 [0.090, 0.118]***	**0.123 [0.111, 0.133]***

Table S3. Clone proportion recovery (H MAE; lower is better). Values are mean ± standard deviation across 20 replicates. Fold-improvement shows how many times lower ClonalGE error is relative to STdeconvolve.

Configuration	ClonalGE	STdeconvolve	Fold↑
Normal	0.0301 ± 0.0107	0.0682 ± 0.0187	2.3×
Low Variance	0.0174 ± 0.0021	0.0463 ± 0.0165	2.7×
High Coverage	0.0191 ± 0.0018	0.0683 ± 0.0241	3.6×

Table S4. Clone-specific gene expression recovery (B MAE, row-normalised; lower is better). Values are mean ± standard deviation across 20 replicates. Fold-improvement shows how many times lower ClonalGE error is relative to STdeconvolve.

Configuration	ClonalGE	STdeconvolve	Fold↑
Normal	0.0015 ± 0.0002	0.0045 ± 0.0018	3.0×
Low Variance	0.0006 ± 0.0001	0.0032 ± 0.0016	5.2×
High Coverage	0.0014 ± 0.0001	0.0045 ± 0.0026	3.2×

Table S5. Wall-clock runtime of ClonalGE and Tumoroscope across all simulation conditions and on real prostate cancer data. Values are mean ± SD (range in parentheses) in minutes, computed over 20 independent replicates for each simulation setup and method, and over 20 independent runs for the real prostate data. All simulated experiments used the same iteration bounds (minimum 10 000, maximum 30 000 iterations) and the same 99.5% Geweke convergence stopping criterion. The elevated mean and standard deviation for Tumoroscope in the *normal* and *low expression variance* conditions reflect slower and more variable convergence in the absence of gene expression information. For ClonalGE 5-chains, five MCMC chains were executed in parallel on separate CPU cores; total wall-clock time therefore scales sub-linearly with the number of chains. Runtimes exceeding ≈60 min in the ClonalGE conditions (visible in the elevated maxima) correspond to runs that required the full 30 000 iterations before the convergence criterion was met. The *high coverage* setup converges faster for all methods because higher read depth produces sharper likelihood gradients. For the real prostate cancer data (294 spots, $K = 4$ clones, $G = 1000$ genes), each run comprised 10 parallel MCMC chains; the substantially longer runtimes relative to simulated data reflect the 20-fold larger number of genes and the higher chain count.

**Method**	***Normal***	***Low expression variance***	***High coverage***
Tumoroscope	37.1 ± 10.2 (16.9–46.2)	41.5 ± 9.7 (16.8–47.1)	19.1 ± 0.2 (18.7–19.6)
ClonalGE (1 chain)	21.9 ± 0.1 (21.7–22.1)	23.7 ± 8.2 (21.6–59.3)	23.7 ± 0.1 (23.3–24.0)
ClonalGE (5 chains)	28.4 ± 13.3 (22.7–60.3)	22.8 ± 0.2 (22.6–23.2)	24.9 ± 0.2 (24.5–25.3)
Real data (prostate cancer, 10 chains per run): 769.8 ± 187.7 min (520.3–1047.4 min), $n = 20$ runs.			

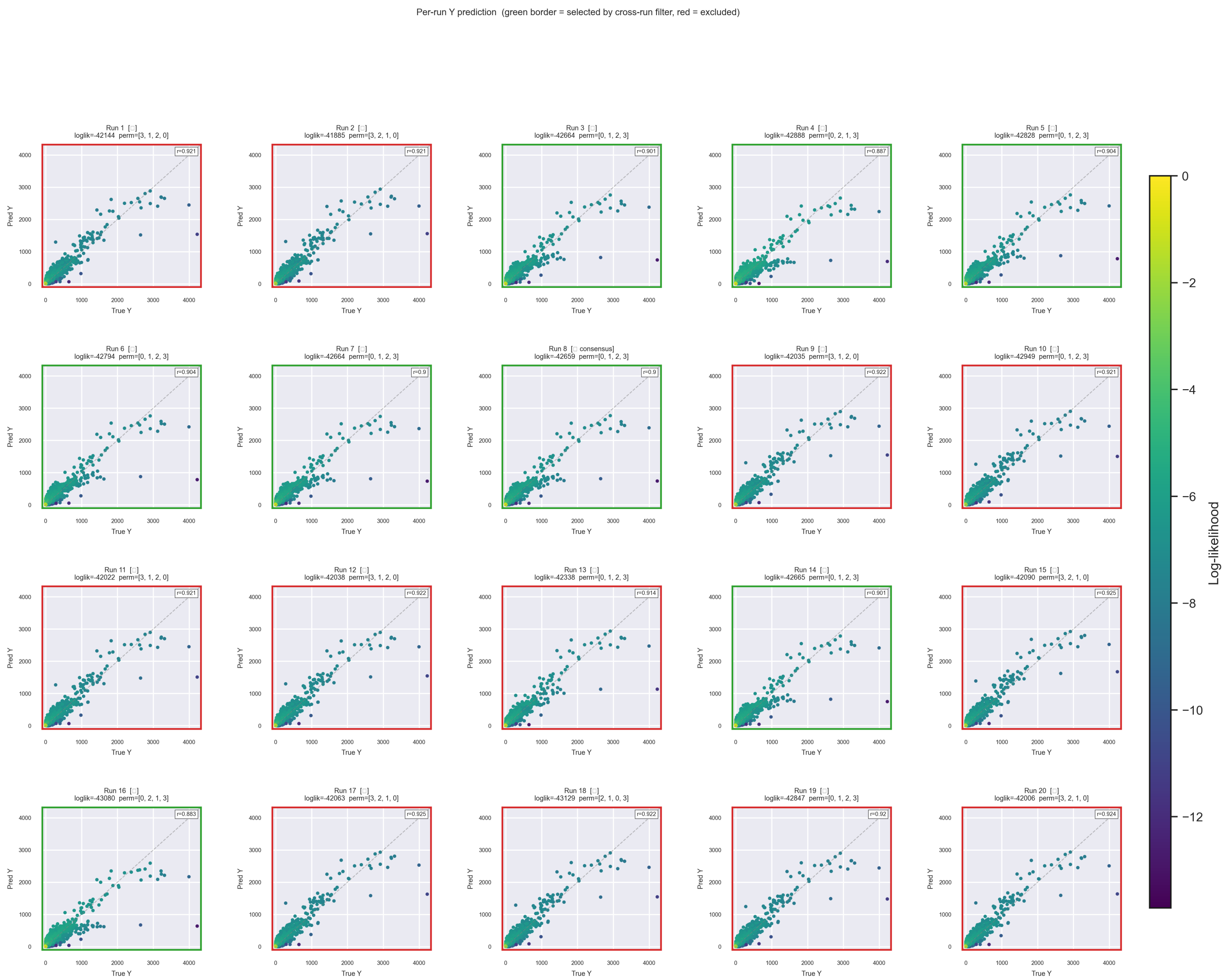

Fig. S5. Per-run predicted versus observed gene expression across all 20 independent runs. For each run, the within-run averaged H, B, and N were used to compute the predicted expression matrix $\hat{Y} = \text{diag}(N) \cdot H \cdot B$, with per-section scaling applied to account for differing sequencing depths across the three tissue sections (SP1, SP2, SP3). Each point represents one gene–spot observation, coloured by the per-observation negative-binomial log-likelihood. The Pearson r between Y_{true} and $\hat{Y}$ is annotated in each panel. Green borders indicate runs selected by the consensus cross-run filter (runs 3, 4, 5, 6, 7, 8, 14, 16); red borders indicate the excluded runs; run 8 is additionally labelled as the consensus reference. All runs achieve $r \approx 0.88$–0.93, confirming that every run fits the observed expression data well regardless of clone-label orientation, and that exclusion of the remaining 12 runs is driven by label switching rather than inferior predictive accuracy.

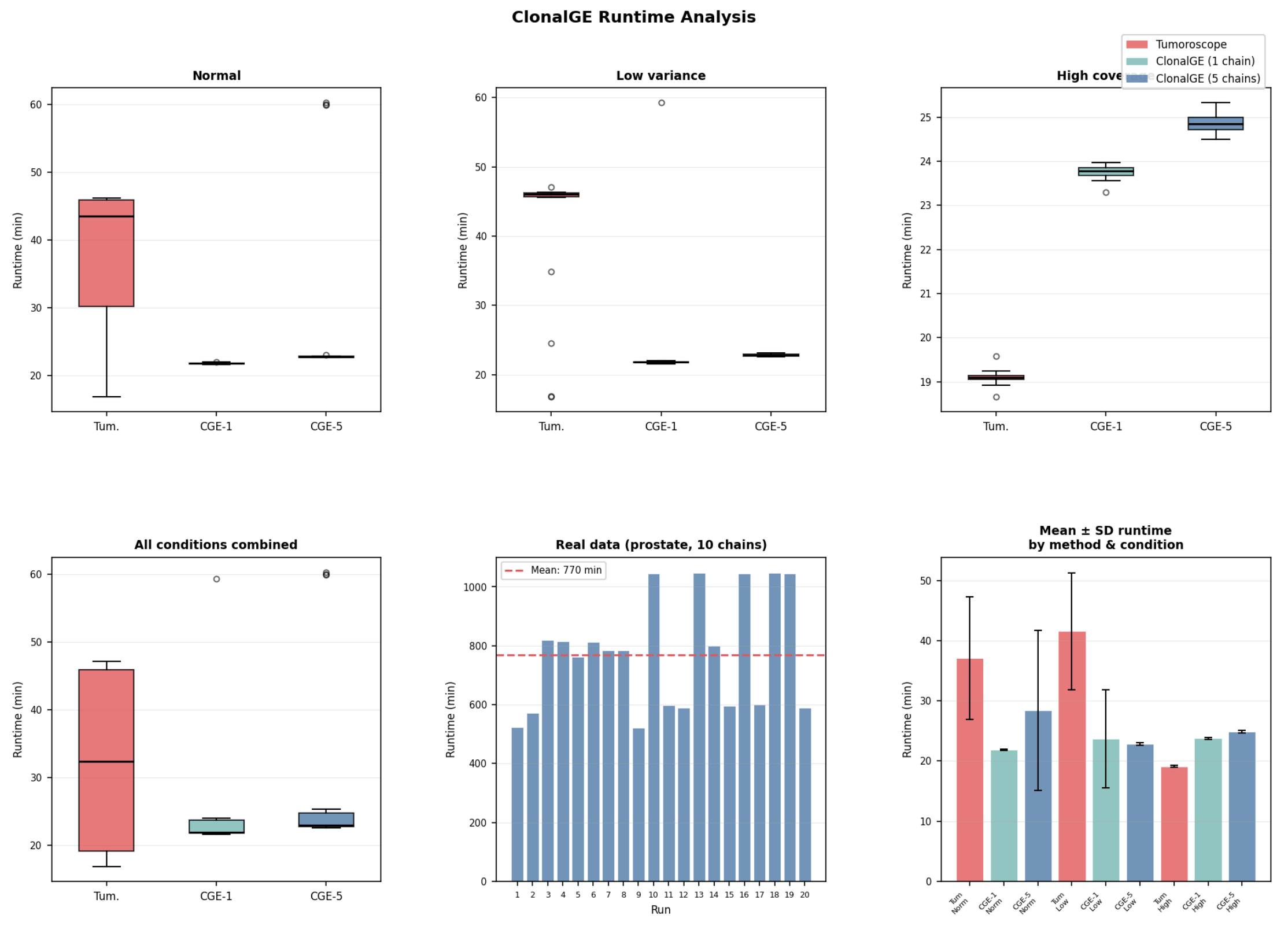

Fig. S6. Runtime distributions for ClonalGE and Tumoroscope. Wall-clock runtimes (minutes) across 20 independent replicates for each simulation configuration and method. Box plots show median, interquartile range, and 1.5×IQR whiskers; individual replicates are overlaid as points. Dashed vertical lines mark the median runtime of Tumoroscope for reference. Outliers in ClonalGE conditions correspond to replicates that ran to the maximum iteration bound (30 000) before the Geweke convergence criterion was satisfied.

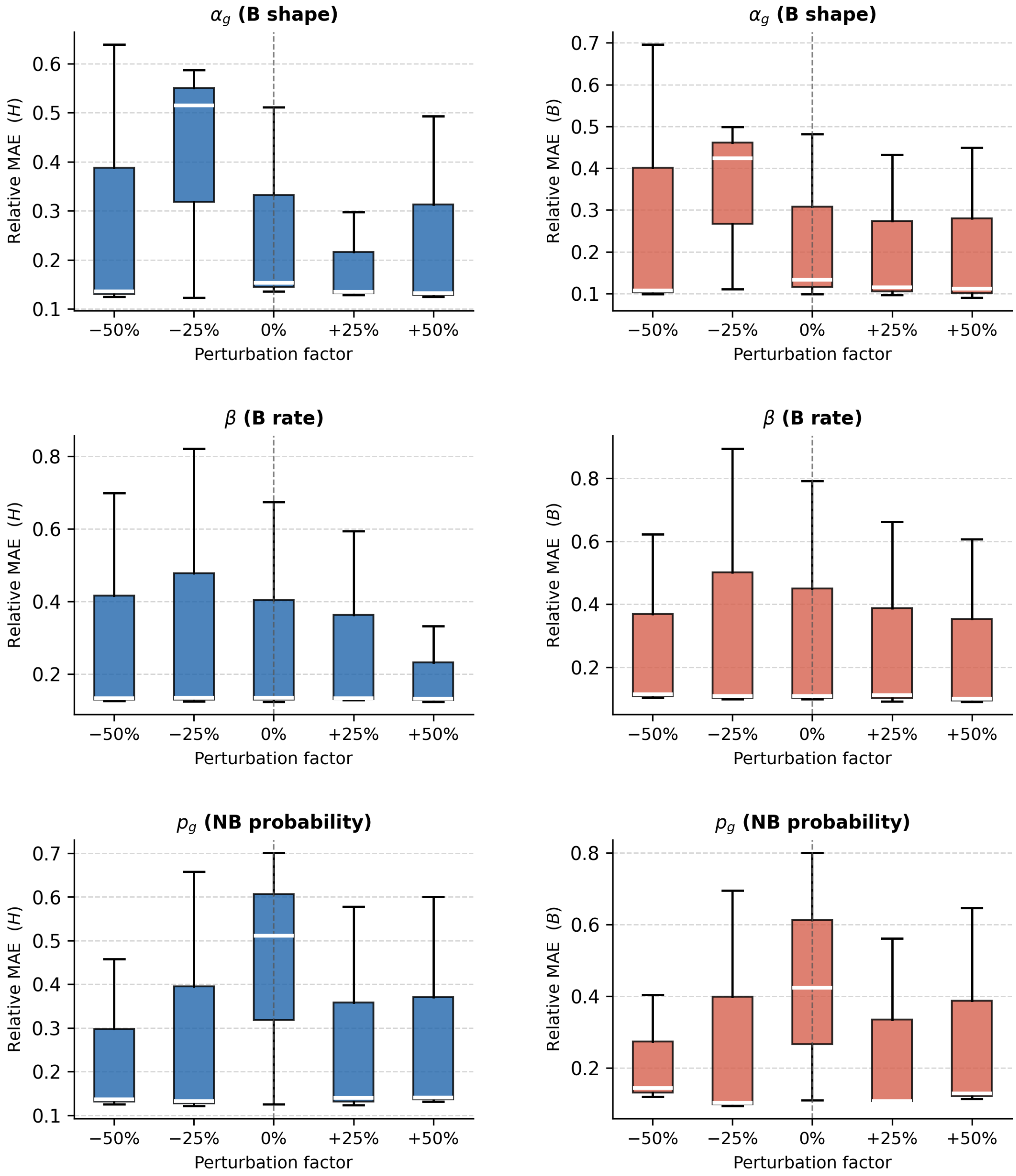

Fig. S7. Supplementary Figure: Hyperparameter sensitivity of ClonalGE. Relative MAE for clone-fraction recovery (H, left) and clonal gene expression recovery (B, right) as a function of hyperparameter perturbation factor applied to α_g (Gamma shape of the B prior; top row), β (Gamma rate of the B prior; middle row), and p_g (Negative Binomial success probability; bottom row). Each boxplot summarises 3 independent simulation replicates of the *normal* configuration; the factor 1.0 (dashed line) corresponds to the true (data-derived) hyperparameter value.

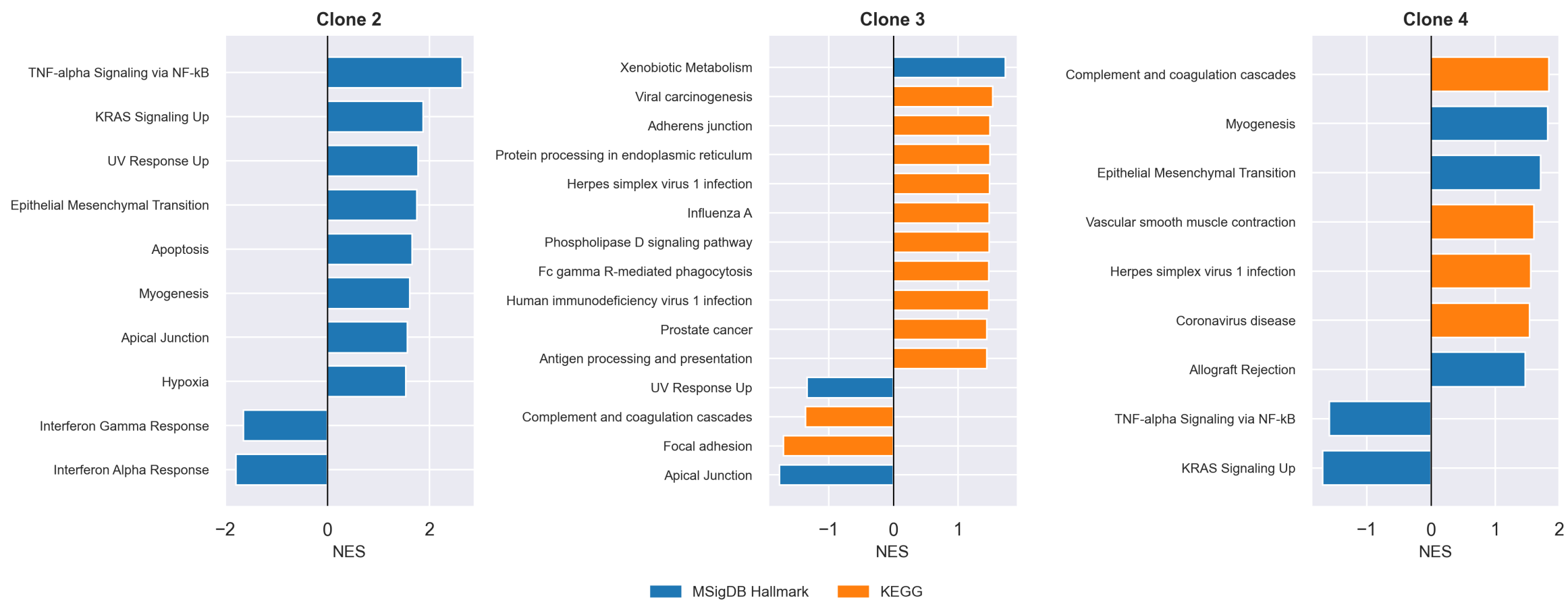

Fig. S8. Supplementary Figure: Gene Set Enrichment Analysis (GSEA) for clone-specific gene expression. Pre-ranked GSEA results for each non-normal inferred clone (panels) using MSigDB Hallmark and KEGG pathway gene sets (colour-coded in the legend). Genes were ranked by the per-clone posterior mean log2 fold-change $\text{score}_k(g) = \mathbb{E}[\log_2(B_{k,g}+\varepsilon_0) \mid \text{Data}] - \frac{1}{|\mathcal{K}'|}\sum_{k'} \mathbb{E}[\log_2(B_{k',g}+\varepsilon_0) \mid \text{Data}]$, where ε_0 is the pseudocount defined in Methods, capturing both direction and magnitude of clone k's differential expression relative to all other clones. Normalised enrichment scores (NES) are shown for gene sets with false-discovery-rate-adjusted $q < 0.25$. Positive NES indicates enrichment among clone-specific over-expressed genes.

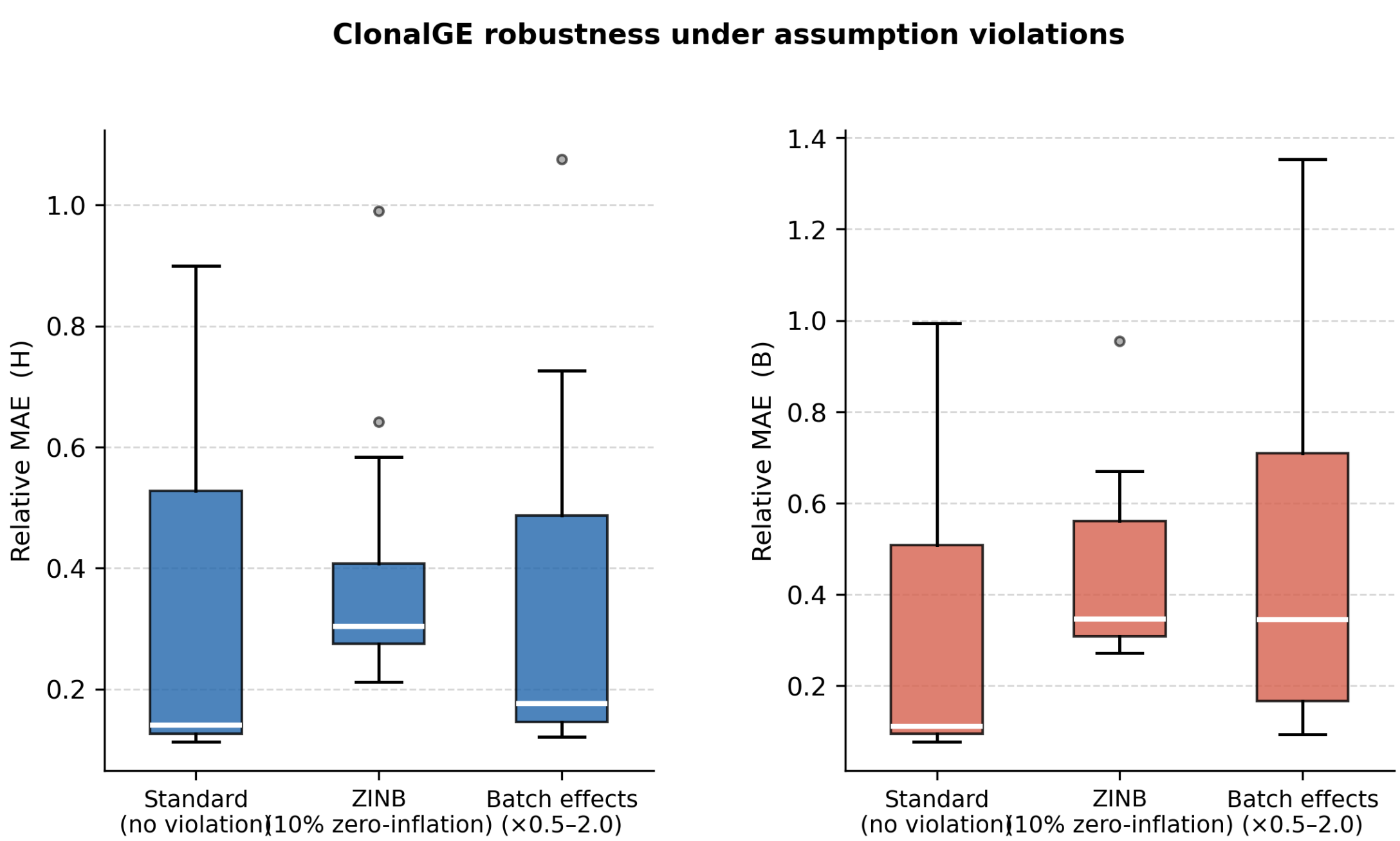

Fig. S9. Supplementary Figure: ClonalGE performance under assumption-violating simulation conditions. Relative MAE for H and B under three conditions: (i) standard simulation (ground truth, no assumption violations), (ii) zero-inflated Negative Binomial gene expression (10% zero-inflation rate), and (iii) section-level batch effects (multiplicative factors drawn from Uniform$(0.5, 2.0)$). Each boxplot shows 20 independent replicates; lower values indicate better inference accuracy. Results demonstrate that ClonalGE remains robust to moderate deviations from the NB distributional assumption and to batch effects of this magnitude.
